# Efficient total synthesis of dehydro-*δ*-viniferin through metal–halogen exchange cyclization†

**DOI:** 10.1039/d5ra00960j

**Published:** 2025-03-11

**Authors:** Jinfang Cao, Qibin Zhu, Zhen Sha, Xiaoxuan Zhou, Ruitong Zhu, Chunsuo Yao

**Affiliations:** a State Key Laboratory of Bioactive Substance and Function of Natural Medicines, Institute of Materia Medica, Chinese Academy of Medical Sciences, Peking Union Medical College Beijing 100050 P. R. China yaocs@imm.ac.cn

## Abstract

Bioactive dehydro-*δ*-viniferin was synthesized through an efficient and practical eight-step route, achieving an overall yield of 27%. The synthesis process involves an intramolecular cyclization and dehalogenation *via* a metal–halogen exchange, producing 3-arylbenzofuran, with the di-iodinated *α*-aryloxyketone serving as the key intermediate. Long reaction times and the use of excess reagent *i*-PrMgCl·LiCl facilitate metal–halogen exchange cyclization and dehalogenation. This synthetic approach, scalable for the production of analogs, was successfully conducted for the first time in multigram quantities.

## Abstract 2

Metal–halogen exchange cyclization and dehalogenation with the di-iodinated *α*-aryloxyketone serving as the key intermediate resulted in a highly efficient total synthesis of dehydro-*δ*-viniferin in eight steps with an overall yield of 27%.

## Graphical abstract

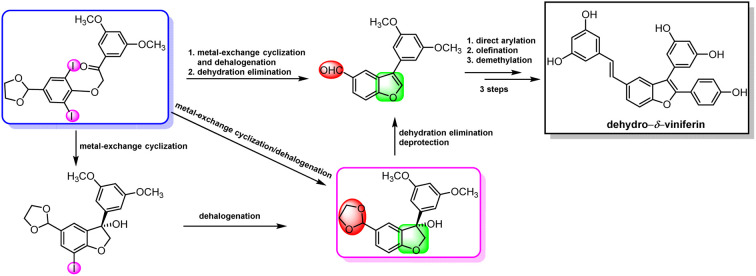

## Introduction

**Fig. 1:**
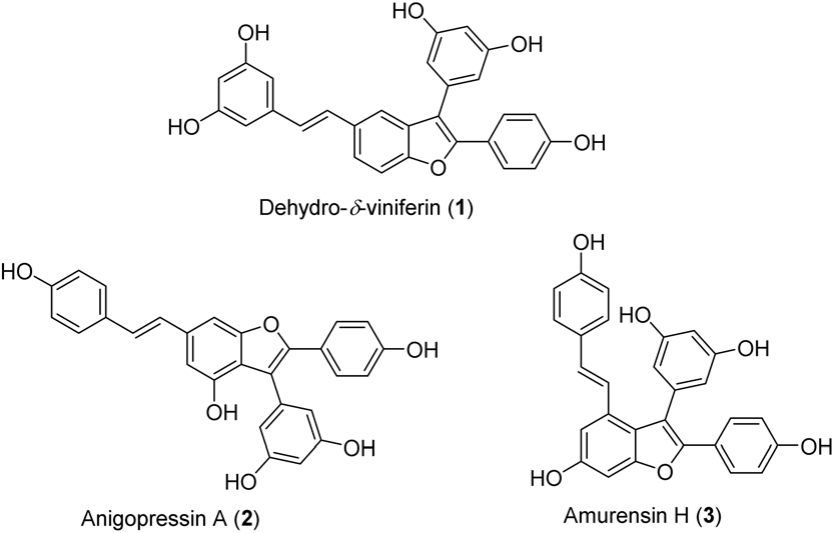
Active stilbene dimers with 2,3-diarylbenzofuran skeleton.

Oligostilbenes, a class of polyphenols derived from resveratrol and its analogs, have attracted considerable interest over the past three decades due to their unique structures and diverse bioactivities.^1–3^ Stilbene dimers with a 2,3-diarylbenzofuran skeleton, such as dehydro-*δ*-viniferin (1), anigopreissin A (2), and amurensin H (3) (Fig. 1), display potent anti-inflammatory, antioxidant, antifungal, antibacterial, and anticancer activities.^3,4^ Resveratrol dimers with a benzofuran skeleton have been recently identified as novel inhibitors of the T3SS (type III secretion system) in *Y. pseudotuberculosis* and *P. aeruginosa*.^5^ Dehydro-*δ*-viniferin (1), a benzofuran derivative of the natural product *δ*-viniferin, has shown substantial inhibitory activity against T3SS, better efficacy in rescuing eukaryotic cells from infection, and strong toxicity toward eukaryotic cells at high concentrations.^5^ Additionally, compound 1 exhibited remarkable antibacterial activity against *S. aureus* and antioxidant properties.^6,7^

Considering their interesting structure and unexplored potential in medicine, several synthetic approaches to these compounds have been reported. Notable strategies include the biotransformation strategy reported by Beneventi *et al.*^7^ and the chemically controlled approaches described by Mattio, Shaw, and, most notably, the Elofsson groups.^6,8,9^ As described by Beneventi *et al.*,^7^ dehydro-*δ*-viniferin 1 was semi-synthesized through a laccase-biocatalyzed dimerization of resveratrol, followed by a dehydrogenation reaction of *δ*-viniferin. Its total synthesis was also realized through a one-pot, two-step Sonogashira cyclization of *o*-iodophenol and 4-ethynylanisole, as reported by Mikael Elofsson *et al.*^9^

In previous work,^10^ the stilbene dimer dehydro-*δ*-viniferin was successfully synthesized in nine steps, with the Amberlyst 15-catalyzed cyclodehydration of *α*-aryloxyketone serving as the key step. However, the lower yield proved to be less effective to some extent. Therefore, developing a facile and practical synthetic route is necessary for further investigating compound 1. This paper reports a highly efficient and operationally convenient route to the dehydro-*δ*-viniferin and its analogs, using metal–halogen exchange cyclization and dehalogenation as the key step, and at a scale that addresses the demands for structure–activity relationship studies.

## Results and discussion

**Scheme 1:**
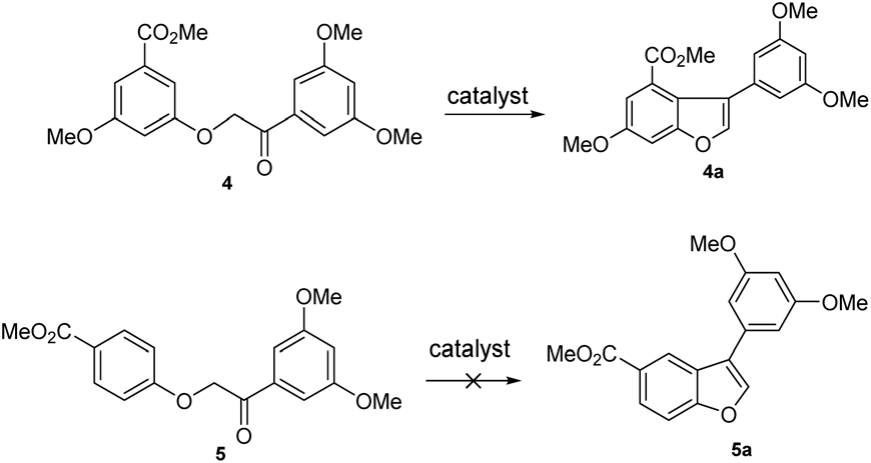
Cyclodehydration of *α*-aryloxyketones 4 and 5.

Previous research demonstrated that the low yield in the construction of 2-arylbenzofuran catalyzed by Amberlyst 15 led to a less efficient synthetic route for the synthesis of compound 1.^10^ Literature and experiment reports revealed that the cyclodehydration reaction of *α*-aryloxyketone (4) was possible,^10–13^ but the cyclodehydration reaction of *α*-aryloxyketone (5) proved to be challenging in the same manner (Scheme 1). The lower reactivity of intermediate (5), which lacks methoxy groups on the aromatic rings, combined with the instability of the ether linkage, resulted in a lower yield during cyclodehydration. Constructing the 2-arylbenzofuran intermediate 5a using the cyclodehydration reaction in this procedure is impractical. Thus, the critical issue that must be addressed lies in achieving the installation of the 2-arylbenzofuran intermediate with a higher yield.

**Scheme 2:**
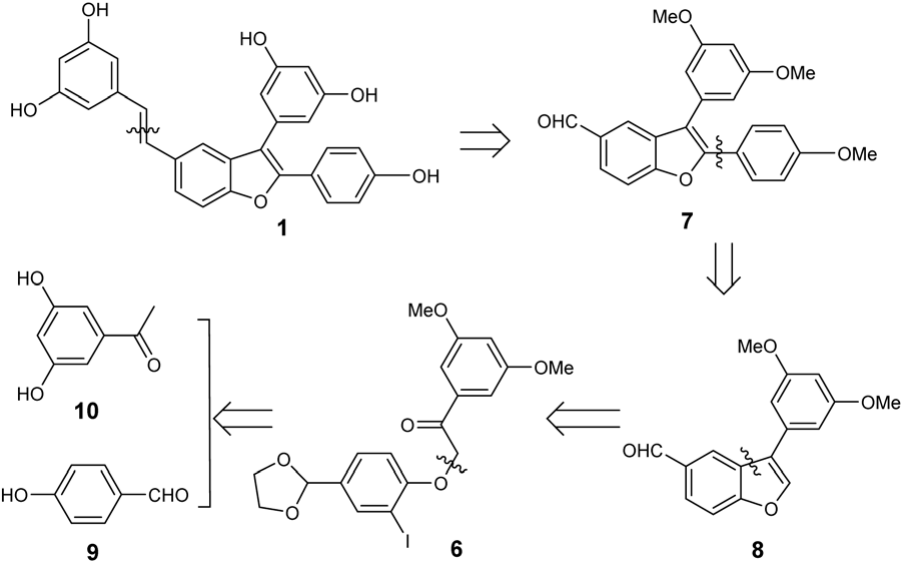
Retrosynthetic analysis of dehydro-*δ*-viniferin (1).

As reported in the literature,^14,15^ benzofuran carboxaldehyde can be generated in high yield *via* a metal–halogen exchange between iodo ketone and methyllithium, followed by treatment with PTSA. Based on these results, this study assumed that metal–halogen exchange using alkyllithium reagents, followed by treatment with PTSA in dichloromethane, could provide a simple and convenient method for synthesizing dehydro-*δ*-viniferin. Accordingly, iodo ketone 6 has been selected as the key synthetic precursor for compound 1, and the retrosynthetic route is designed and outlined in Scheme 2. The target compound 1 could be prepared through the olefination of benzofuran aldehyde 7. This aldehyde could be prepared *via* a transition-metal-catalyzed direct C–H activation of benzofuran 8, followed by intermolecular coupling with an appropriate aryl bromide. The requisite 8 is expected to be derived from iodo ketone 6, which can be conveniently prepared from commercially available 4-hydroxybenzaldehyde (9) and 3,5-dihydroxyacetophenone (10).

**Scheme 3:**
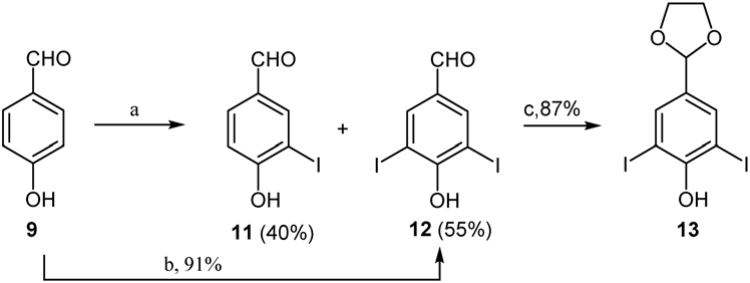
Iodination and protection of 4-hydroxybenzaldehyde 9. Reagents and conditions: (a) NIS (1.2 eq.), TFA, r.t., 6 h, 40% (11), 55% (12); (b): NIS (2.2 eq.), TFA, r.t., 6 h, 91%; (c) TMSCl, DCM, ethylene glycol, reflux, 18 h, 87%.

As outlined in Scheme 3, the synthesis began with the preparation of the required 3-iodo-4-hydroxyaldehyde (11). Following the iodination reaction described by Castanet *et al.*,^16,17^ iodination of 4-hydroxybenzaldehyde (9) was conducted in the presence of NIS (1.2 eq.) and trifluoroacetic acid. However, the reaction produced an inseparable mixture of the monoiodinated benzaldehyde 11 and di-iodinated benzaldehyde (12) in a 43 : 57 ratio, instead of the target product 11. Thus, the molar ratio of the substrate to reagent was altered from 1 : 1.2 to 1 : 1.8, or 1 : 2.2, to improve the reaction efficiency. As a result, the di-iodinated product 12 was obtained in 91% yield at a ratio of 1 : 2.2, though the monoiodinated product 11 was still unsuccessfully isolated in higher yield. Theoretically, subsequent dehalogenation reactions at the benzofuran ring after cyclization can be easily conducted; thus, di-iodinated product 12 remains a viable intermediate for the designed synthetic route. Therefore, this strategy is advantageous compared to using the monoiodinated product as the key intermediate.^15,18^ Afterward, protection of the aldehyde 12 using TMSCl and ethylene glycol under refluxing dichloromethane conditions successfully yielded the glycol acetal 13 in 87% yield.^19^

**Scheme 4:**
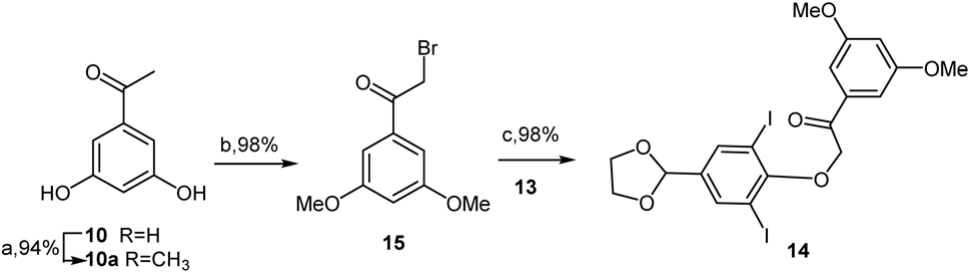
Synthesis of di-iodinated *α*-aryloxyketone 14. Reagents and conditions: (a) CH_3_I, K_2_CO_3_, acetone, r.t., 24 h, 94%; (b) TBABr_3_, THF-methanol, reflux, 3 h, 98%; (c) K_2_CO_3_, acetone, reflux, 3 h, 98%.

Using prepared glycol acetal 13, this study focused on constructing the di-iodinated *α*-aryloxyketone 14, which was achieved through the etherification of phenol 13 with 2-bromo-3,5-dimethoxyacetophenone (15) in boiling acetone in the presence of potassium carbonate, which resulted in a 98% yield. Compound 15 was easily synthesized from commercially available 3,5-dihydroxyacetophenone 10*via* methylation with methyl iodide in acetone, followed by *α*-bromination with TBABr_3_ in THF and methanol (Scheme 4).^11–13^

**Scheme 5:**
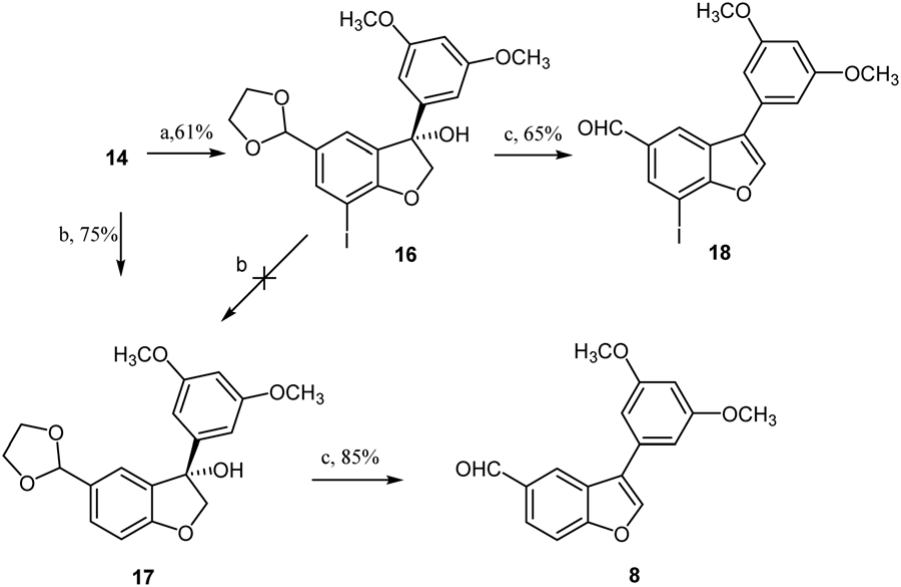
Metal–halogen exchange cyclization, dehalogenation and dehydration reaction of di-iodinated *α*-aryloxyketone 14. Reagents and conditions: (a) *i*-PrMgCl·LiCl (3.5 eq.), THF, −78 °C, 0.5 h, 61%; (b) *i*-PrMgCl·LiCl (3.5 eq.), THF, −78 °C, 2 h, 75%; (c) *p*-TsOH, DCM, r.t., 2 h, 65% or 85%.

After preparing the di-iodinated *α*-aryloxyketone 14, the installation of the key intermediate 3-arylbenzofuran 8*via* a metal–halogen exchange reaction commenced (Scheme 5). Previous reports indicated that metal–halogen exchange with alkyllithium is faster than with a carbonyl group.^20,21^ Hence, the approach described by Kraus and Kim was initially explored, and compound 14 was treated with three equivalents of methyllithium at −78 °C in THF.^14,15^ Unfortunately, this reaction resulted in a complex mixture, with no detection of the expected monoiodinated benzofuran 16, as indicated by TLC. Furthermore, using butyllithium instead of methyllithium, the metal–halogen exchange reaction of 14 in THF generated only the dehalogenated starting compound, rather than the target compound 16. Intensive exploration of the reaction conditions surprisingly revealed that exposure of 14 to 3.5 equivalents *i*-PrMgCl·LiCl in THF at −78 °C for 0.5 h, following the reported procedure,^22,23^ generated the desired monoiodinated benzofuran 16 in 61% yield. However, when the reaction time was extended to 2 h, intermediate 14 was unexpectedly directly converted into the dehalogenated alcohol 17 in 75% yield, instead of the expected monoiodinated product 16. This finding indicates that a longer reaction time facilitates the intramolecular cyclization and dehalogenation simultaneously. Further exploration indicates that the longer reaction time and using more than 3.5 equivalent reagents (*i*-PrMgCl·LiCl) were crucial for generating dehalogenated product 17. By contrast, when monoiodinated alcohol 16 was treated under the same conditions, the expected dehalogenated product 17 remained undetected in the product mixture, as indicated by TLC.

**Scheme 6:**
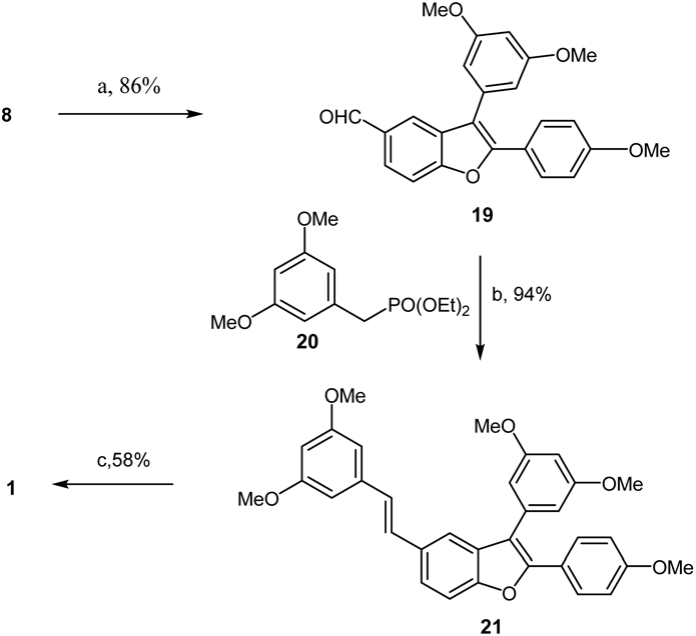
Synthesis of dehydro-*δ*-viniferin 1. Reagents and conditions: (a) 4-bromoanisole, Pd(OAc)_2_, Cs_2_CO_3_, PCy_3_·HBF_4_, DMA, 140 °C, 86%; (b) *t*-BuOK, THF, 0 °C – r.t., 94%; (c) BBr_3_, DCM, −45–0 °C, 58%.

With the efficient construction of alcohol 17 realized, the path was cleared for completing the synthesis of aldehyde 8, as illustrated in Scheme 5. Based on previous work reported in the literature,^14,15,24^ treatment of alcohol 17 or monoiodinated alcohol 16 in dichloromethane with PTSA (15% mol) at room temperature resulted not only in dehydration but also in the deprotection of the dioxolane, generating the desired aldehyde 8 or monoiodinated aldehyde 18 in 85% and 65% yields, respectively. Subsequently, aldehyde 8 was reacted with 4-bromoanisole in the presence of Pd(OAc)_2_ (0.2 equiv.) and Cs_2_CO_3_ (2 equiv.) at 140 °C in DMA, producing aldehyde 19 in 86% yield. The Horner–Wadsworth–Emmons-type olefination of 19 with diethyl 3,5-dimethoxybenzylphosphonate (20) in THF at 0 °C to room temperature resulted in the formation of permethylated dehydro-*δ*-viniferin (21) in 94% yield. Finally, treatment 21 with BBr_3_ in dichloromethane at −45 °C to room temperature for 15 h successfully yielded the target compound, dehydro-*δ*-viniferin, in 58% yield (Scheme 6).

## Conclusions

Overall, this study developed an efficient, concise, and practical total synthetic route to the active resveratrol dimer, dehydro-*δ*-viniferin, in eight steps, with an overall yield of 27% from 3,5-dihydroxyacetophenone. A metal–halogen exchange cyclization and dehalogenation was used to construct the key intermediate, 3-arylbenzofuran, with the di-iodinated *α*-aryloxyketone serving as the key intermediate. Extended reaction times and excess reagent *i*-PrMgCl·LiCl facilitate metal–halogen exchange cyclization and dehalogenation. The starting materials are readily available, and the reaction is operationally convenient. This synthetic approach, which is scalable for analogs, was successfully applied to multi-gram quantities for the first time, providing the necessary amounts for further studies on the bioactivity of dehydro-*δ*-viniferin. With sufficient quantities currently available, modifications of dehydro-*δ*-viniferin are presently underway.

## Experimental

Synthetic procedures and full characterization data are provided in the ESI.†

## Data availability

The data supporting this article have been included as a part of the ESI.† ESI† includes synthetic procedures, compound characterization, ^1^H, and ^13^C NMR, and HRMS spectra (PDF).

## Author contributions

Jinfang Cao and Qibin Zhu performed chemical synthesis and characterization data of all compounds. Chunsuo Yao designed and supervised the study. Qibin Zhu, Jinfang Cao and Chunsuo Yao wrote the original draft of the manuscript. Zhen Sha, Xiaoxuan Zhou and Ruitong Zhu reviewed and edited the manuscript. All authors have read and agreed to the published version of the manuscript.

## Conflicts of interest

There are no conflicts to declare.

## Supplementary Material

RA-015-D5RA00960J-s001
