# Supplementary material for: Efficient total synthesis of dehydro-δ-viniferin through metal–halogen exchange cyclization
Source: RSC Adv. 2025 Mar 11;15(10):7738–41. doi: 10.1039/d5ra00960j (PMC11895512; doi:10.1039/d5ra00960j)
Supplement: RA-015-D5RA00960J-s001 [file RA-015-D5RA00960J-s001.pdf]

Supplementary Information (SI) for RSC Advances.
This journal is © The Royal Society of Chemistry 2025

Electronic Supplementary Materials

Efficient total synthesis of dehydro-*δ*-viniferin through metal-halogen exchange cyclization†

Jinfang Cao,‡ Qibin Zhu,‡ Zhen Sha, Xiaoxuan Zhou, Ruitong Zhu and Chunsuo Yao*

† State Key Laboratory of Bioactive Substance and Function of Natural Medicines, Institute of Materia Medica, Chinese Academy of Medical Sciences & Peking Union Medical College, Beijing 100050, P. R. China

*Fax: +86-10-6301-7757; e-mail: yaocs@imm.ac.cn

‡ Jinfang Cao and Qibin Zhu contributed equally to this work.

Content

Section 1. General Experimental ······ S2

Section 2. Experimental for the synthesis of dehydro-*δ*-viniferin (1) ······ S2

Section 3. Spectra in the total synthesis of dehydro-*δ*-viniferin (1) ······ S11

3.1 Spectra of 11 ······ S11

3.2 Spectra of 12 ······ S12

3.3 Spectra of 13 ······ S13

3.4 Spectra of 10a ······ S15

3.5 Spectra of 15 ······ S16

3.6 Spectra of 14 ······ S18

3.7 Spectra of 16 ······ S19

3.8 Spectra of 17 ······ S21

3.9 Spectra of 8 ······ S22

3.10 Spectra of 18 ······ S24

3.11 Spectra of 19 ······ S25

3.12 Spectra of 21 ······ S27

3.13 Spectra of 1 ······ S28

Section 1. General Experimental

Unless otherwise mentioned, reagents and solvents were purchased from commercial sources. Unless otherwise stated, all commercially available reagents were used without further purification. Dry solvents were purchased from commercial sources. Acetone and toluene were dried by $MgSO_4$. HR-MS-ESI and ESI spectra were measured using an AccuToFCS JMST100CS spectrometer (Agilent Technologies, Ltd, Santa Clara, CA, USA). Column chromatography (CC) was performed with silica gel (200-300 mesh, Qingdao Marine Chemical Inc. Qingdao, China). The reaction prcess was monitored by TLC. TLC was conducted with glass precoated silica gel GF254 plates (Qingdao Marine Chemical, Inc., Qingdao, China). Compound spots were visualized under UV light. ^{1}H and ^{13}C NMR spectra for all compounds were recorded at 400 MHz (Zhongke-Niujin, Qone), 500 MHz (Bruker Corporation, Karlsruhe, Germany), 500 MHz (JEOL, JNM-ECZ500R, Japan), and 100/125/125 MHz, respectively. The chemical shifts are reported in ppm and yields refer to isolated yields after purification.

Section 2. Experimental for the synthesis of dehydro-*δ*-viniferin

4-Hydroxy-3-iodobenzaldehyde (11) and 4-hydroxy-3,5-diiodobenzaldehyde (12)

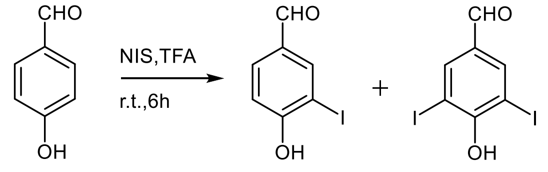

To a solution of 4-hydroxybenzaldehyde **9** (12.2 g, 100.00 mmol) in 150 ml TFA was added 27.5 g NIS (120.00 mmol) in small portions, and the reaction mixture was stirred for 6 h at ambient temperature. After completion of the reaction, the iodinated products were poured into 200 ml ice water and extracted with dichloromethane (250 ml × 3). The combined organic extracts were washed with 10% $Na_2S_2O_3$, brine, dried over anhydrous Na_2SO_4 and filtered, the filtrate wan then concentrated under reduced pressure to gave a mixture, which was purified by flash column chromatography on silica gel (200-300 mesh) to afford dark red amorphous powders **11** (9.8 g, 40%) and **12** (20.5 g, 55%) respectively.

To a solution of 4-hydroxybenzaldehyde **9** (22.4 g, 200.0 mmol) in 600 ml TFA, 110.0 g NIS (440 mmol) was added in batch. The resulting mixture was stirred for 6 h at ambient temperature. After completion, the mixture was cooled to 0 °C, quenched

with saturated $NaHCO_3$, and extracted with DCM. The combined organic layer was washed with 10% $Na_2S_2O_3$, brine, dried over anhydrous Na_2SO_4, and concentrated in vacuo untill precipitation appears. The precipitation was filtered and the filter cake was dried to afford a dark red amorphous powder **12** (62.5 g, 91%).

4-Hydroxy-3-iodobenzaldehyde (**11**): dark red amorphous powder, mp 96.8–99.8 114116 °C; ^{1}H NMR (500 MHz, acetone-d$_6$): δ 10.20 (brs, 1H), 9.81 (s, 1H), 8.28 (d, J = 2.5 Hz, 1H), 7.80 (dd, J = 2.5 Hz, J = 10.5 Hz, 1H), 7.11 (d, J = 10.5 Hz, 1H). ^{13}C NMR (125 MHz, acetone-d$_6$): δ 190.00, 162.71, 142.29, 132.26 (2 × C), 116.03, 84.72 . (+)-HRESI-MS: m/z 246.9255 [M-H]$^-$ (Calcd for $C_7H_4O_2I$: 246.9262).

4-Hydroxy-3,5-diiodobenzaldehyde (**12**): dark red amorphous powder, mp 180.3–184.0 °C; ^{1}H NMR (400 MHz, acetone-d$_6$): δ 9.80 (s, 1H), 8.31 (s, 2H). ^{13}C NMR (125 MHz, acetone-d$_6$): δ 188.99, 161.08, 142.00 (2 × C), 133.80, 84.32 (2 × C). (+)-HRESI-MS: m/z 372.8231 [M-H]$^-$ (Calcd for $C_7H_4O_2I_2$: 372.8228).

4-(1,3-Dioxolan-2-yl)-2,6-diiodophenol (13)

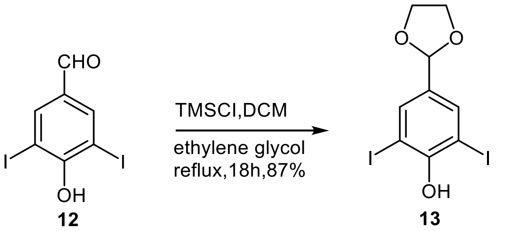

A solution of aldehyde **12** (30.0 g, 109.54 mmol), ethylene glycol (54.7 g, 876.36 mmol), and TMSCl (71.4 g, 657.27 mmol) in 400 mL dichloromethane was refluxed for 18 h . After completion of the reaction, the mixture was cooled to room temperature, quenched with saturated aqueous $NaHCO_3$, and extracted with dichloromethane. The combined organic extracts were dried over $MgSO_4$, filtered and concentrated in vacuo. The crude residue was then purified by silica gel (200-300 mesh) column chromatography (petroleum ether : ethyl acetate : DCM = 15 : 1 : 2, v/v) to afford a white amorphous powder **13** (39.8 g) in 87% yield.

4-(1,3-Dioxolan-2-yl)-2,6-diiodophenol (**13**): white amorphous powder, mp 104.0–106.9 °C; ^{1}H NMR (400 MHz, acetone-d$_6$): δ 7.84 (s, 2H), 5.65 (s, 1H), 4.08 (m, 2H), 3.97 (m, 2H). ^{13}C NMR (125 MHz, acetone-d$_6$): δ 156.58, 138.84 (2 × C), 135.62, 102.10, 83.84 (2 × C), 65.93 (2 × C), 31.12. (+)-HRESI-MS: m/z 416.8492 [M-H]$^-$ (Calcd for $C_9H_7O_3I_2$: 416.8490).

3,5-Dimethoxyacetophenone (10a)

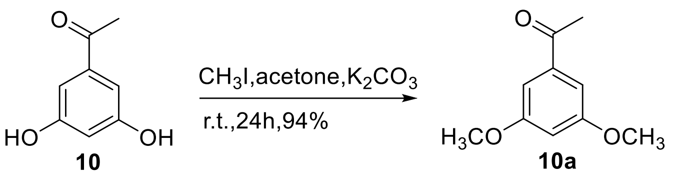

To a solution of 3,5-dihydroxyacetophenone (50.0 g, 328.95 mmol) in 500 ml acetone, K_2CO_3 (181.6 g, 1315.79 mmol) was added and the reaction mixture was stirred for 15 min before 186.7 g iodomethane (1315.79 mmol) was added slowly in batches. The resulting suspension was then stirred at room temperature for 24 h. After completion of the reaction, the mixture was filtered by celite and the filter cake was washed with acetone. The combine filtrate was evaporated in vacuo to give 3,5-dimethoxyacetophenone (**10a**) (55.6 g, 94%) as a dark oil.

3,5-Dimethoxyacetophenone (**10a**): dark oil. ^{1}H NMR (400 MHz, $CDCl_3$): δ 7.26 (d, J = 2.4 Hz, 2H), 6.82 (t, J = 2.4 Hz, 1H), 4.00 (s, 6H), 2.74 (s, 3H). ^{13}C NMR (125 MHz, $CDCl_3$): δ 197.89, 160.94 (2 × C), 139.15, 106.24 (2 × C), 105.41, 55.67 (2 × C), 20.81. (+)-ESI-MS: m/z 181.1 $[M+H]^+$, 203.0 $[M+Na]^+$, 219.0 $[M+K]^+$.

2-Bromo-3,5-dimethoxyacetophenone (15)

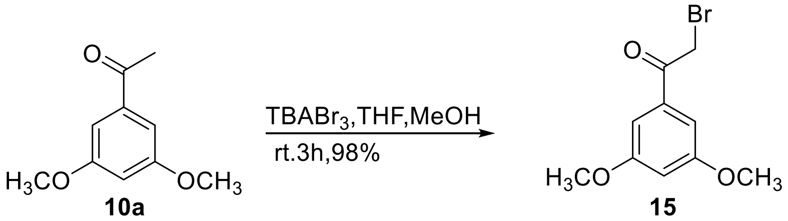

A solution of $TBABr_3$ (79.6 g, 165.00 mmol) in 120 ml THF was added to a stirred solution of 3,5-dimethoxyacetophenone **10a** (27.0 g, 150.00 mmol) in THF (360 ml) and methanol (210 ml). The resulting solution was stirred for 3 h at room temperature. After completion, the reaction mixture was evaporated in vacuo. The residue was redissolved in 600 ml ethyl acetate, the mixture was washed with water, dried over Na_2SO_4, and concentrated in vacuo to afford compound **15** (37.9 g, yield 98%) as a dark red powder.

2-Bromo-3,5-dimethoxyacetophenone (**15**): dark red powder. mp 54.3–57.1 °C; ^{1}H NMR (400 MHz, $CDCl_3$): δ 7.26 (d, J = 2.4 Hz, 2H), 6.85 (t, J = 2.4 Hz, 1H), 4.58 (s, 2H), 4.00 (s, 6H). ^{13}C NMR (125 MHz, $CDCl_3$): δ 191.14, 161.12 (2 × C), 135.90, 106.81 (2 × C), 106.28, 55.77 (2 × C), 31.12. (+)-ESI-MS: m/z 281.0 $[M+Na]^+$.

2-(4-(1,3-Dioxolan-2-yl)-2,6-diiodophenoxy)-1-(3,5-dimethoxyphenyl)ethan-1-one (14)

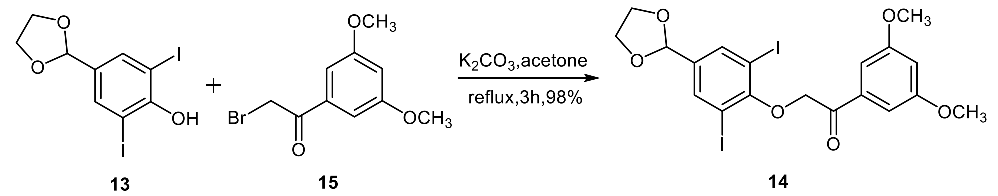

A stirred solution of **13** (42.0 g, 100.00 mmol) and **15** (30.0 g, 115.00 mmol) in acetone (700 ml) was added powdered K_2CO_3 (27.6 g, 200.00 mmol) in batch. The mixture was stirred at room temperature for 2 h before refluxed for 3 h under argon atmosphere. TLC detection demonstrated a completion of the reaction. The mixture was filtered through celite and washed with acetone. The organic layer was concentrated in vacuo. The remaining residue was purified by silica gel (200-300 mesh) column chromatography (petroleum ether : ethyl acetate: dichloromethane = 12 : 1 : 2, v/v) to afford compound **14** (58.4 g, 98%) as a white amorphous powder.

2-(4-(1,3-Dioxolan-2-yl)-2,6-diiodophenoxy)-1-(3,5-dimethoxyphenyl)ethan-1-one (**14**): white amorphous powder. mp 144.9–146.1 °C; ^{1}H NMR (500 MHz, acetone-d_6): δ 7.94 (s, 2H), 7.24 (d, J = 2.4 Hz, 1H), 6.77 (t, J = 2.4 Hz, 1H), 5.72 (s, 1H), 5.36 (s, 1H), 4.11 (m, 2H), 4.00 (m, 2H), 3.86 (s, 6H). ^{13}C NMR (125 MHz, acetone-d_6): δ 192.40, 162.16, 158.78, 140.03, 156.58, 138.84 (2 × C), 135.62, 102.10, 83.84 (2 × C), 65.93 (2 × C), 31.12. (+)-HRESI-MS: m/z 595.9282 [M+H]$^+$ (Calcd for $C_{19}H_{19}O_6I_2$: 596.9266).

3-(3,5-Dimethoxyphenyl)-5-(1,3-dioxolan-2-yl)-7-iodo-2,3-dihydrobenzofuran-3-ol (16)

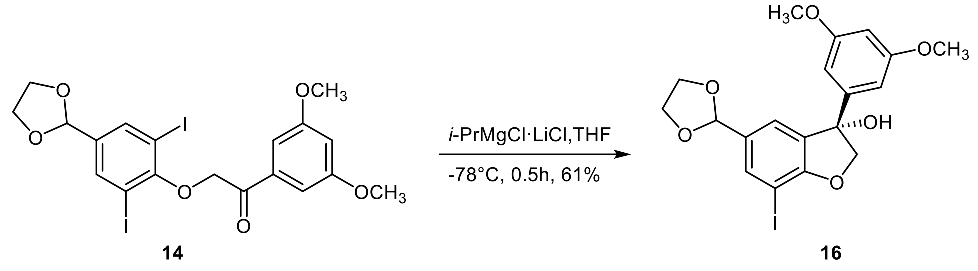

Compound **14** (1.0 g, 1.68 mmol) in dry THF (15 ml) was added slowly to a solution (4.5 ml) of *i*-PrMgCl·LiCl (5.03 mmol, 1.30 mol/l in THF) in THF at –78 °C under argon atmosphere. The mixture was stirred for 0.5 h, the saturated aqueous NH_4Cl (10 ml) was then added to quench the reaction. The resulting mixture was extracted with EtOAc, the combined organic layer was dried over Na_2SO_4, filtered through celite and concentrated in vacuo. The residue was separated by silica gel (200-300 mesh)

column chromatography using petroleum ehter : ethyl acetate : dichloromethane (10 : 1 : 2, v/v) as eluent to afford compound **16** (481 mg) as a light yellow oil in 61% yield.

3-(3,5-Dimethoxyphenyl)-5-(1,3-dioxolan-2-yl)-7-iodo-2,3-dihydrobenzofuran-3-ol (**16**): light yellow oil; ^{1}H NMR (500 MHz, acetone-d$_6$): δ 7.73 (s, 1H), 7.15 (s, 1H), 6.66 (s, 2H), 6.44 (s, 1H), 5.62 (s, 1H), 5.38 (s, 1H), 4.71 (d, J = 10.0 Hz, 1H), 4.65 (d, J = 10.6 Hz, 1H), 4.04 (m, 2H), 3.92 (m, 2H), 3.76 (s, 6H). ^{13}C NMR (125 MHz, acetone-d$_6$): δ 162.30, 161.80 (2 × C), 147.23, 138.14 (2 × C), 134.66, 134.24, 124.23, 105.25 (3 × C), 103.31, 99.71, 87.03, 65.86 (2 × C), 55.63 (2 × C). (+)-HRESI-MS: m/z 493.0118 [M+Na]$^+$ (Calcd for $C_{19}H_{19}O_6INa$: 493.0119).

3-(3,5-Dimethoxyphenyl)-5-(1,3-dioxolan-2-yl)-2,3-dihydrobenzofuran-3-ol (17)

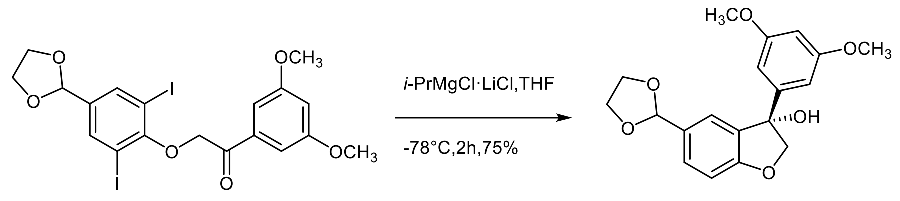

Compound **14** (15.0 g, 25.17 mmol) in dry THF (350 ml) was added slowly to a solution (68 ml) of *i*-PrMgCl·LiCl (88.12 mmol, 1.30 mol/l in THF) in THF at -78°C under argon atmosphere. The reaction mixture was stirred for 2h and the saturated aqueous NH_4Cl (5 ml) was then added to quench the reaction. After that, the mixture was extracted with EtOAc, the combined organic layer was dried over Na_2SO_4, filtered through celite and concentrated in vacuo consecutively. The remaining residue was separated by silica gel (200-300 mesh) column chromatography using petroleum ehter : ethyl acetate : dichloromethane (10 : 1 : 2, v/v) as eluent to afford compound **17** (6.5 g) as a light yellow oil in 75% yield.

3-(3,5-Dimethoxyphenyl)-5-(1,3-dioxolan-2-yl)-2,3-dihydrobenzofuran-3-ol (**17**): light yellow oil. ^{1}H NMR (500 MHz, acetone-d$_6$): δ 7.35 (d, J = 10.0 Hz, 1H), 7.16 (s, 1H), 6.88 (d, J = 10.0Hz, 1H), 6.65 (s, 2H), 6.42 (t, J = 1.6 Hz, 1H), 5.61 (s, 1H), 5.23 (s, 1H), 4.64 (d, J = 10.0 Hz, 1H), 4.60 (d, J = 10.0 Hz, 1H), 4.03(m, 2H), 3.91(m, 2H), 3.75 (s, 6H). ^{13}C NMR (125 MHz, acetone-d$_6$): δ 162.30, 161.79 (2 × C), 147.23, 138.13, 134.63, 134.24, 124.22, 105.24 (2 × C), 103.30, 99.69, 87.01, 83.58, 74.28, 65.85, 65.82, 55.62 (2 × C). (+)-HRESI-MS: m/z 367.1151 [M+Na]$^+$ (Calcd for $C_{19}H_{20}O_6Na$: 367.1152).

3-(3,5-Dimethoxyphenyl)benzofuran-5-carbaldehyde (8)

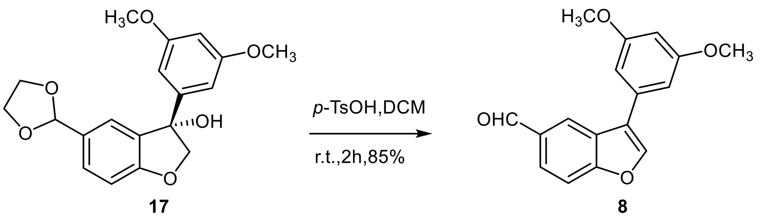

p-TsOH (1.8 g, 4.36 mmol) was added to a stirred solution of compound **17** (10.0 g, 29.06 mmol) in dry dichloromethane (100 ml), the mixture was allowed to stir at room temperature for 2 h. After completion of the reaction, 5 ml water was added and the resulted suspension was extracted with EtOAc. The organic layer was combined, dried over Na_2SO_4, filtered and concentrated under reduced pressure. The remaining residue was purified by silica gel (200-300 mesh) column chromatography with petroleum ehter : ethyl acetate : dichloromethane (20 : 2 : 5, v/v) as eluent to afford compound **8** (7.0 g) as a white amorphous powder in 85% yield.

3-(3,5-Dimethoxyphenyl)benzofuran-5-carbaldehyde (**8**): white amorphous powder. mp 93.6–96.5 °C; ^{1}H NMR (500 MHz, acetone-d$_6$): δ 10.17 (s, 1H), 8.51 (s, 1H), 8.31 (s, 1H), 7.98 (d, J = 9.5 Hz, 1H), 7.78 (d, J = 8.5 Hz, 1H), 6.90 (s, 2H), 6.57 (s, 1H), 3.88 (s, 6H). ^{13}C NMR (125 MHz, acetone-d$_6$): δ 191.60, 161.59, 158.89 (2 × C), 144.15, 133.01, 132.79, 126.78, 125.17, 124.33, 122.68, 112.53, 105.49 (2 × C), 99.55, 54.89 (2 × C). (+)-HRESI-MS: *m/z* 283.0966 [M+H]$^+$ (Calcd for $C_{17}H_{15}O_4$: 283.0965).

3-(3,5-Dimethoxyphenyl)-7-iodobenzofuran-5-carbaldehyde (18)

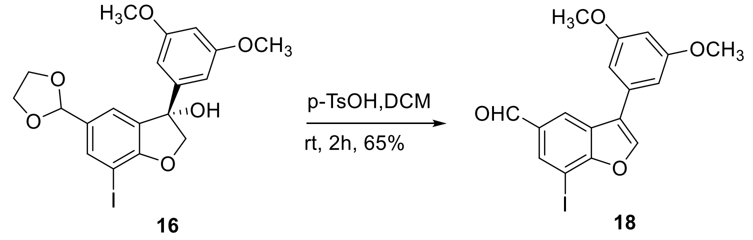

p-TsOH (11 mg, 0.07 mmol) was added to a stirred solution of compound **16** (200 mg, 0.43 mmol) in dry dichloromethane (25 ml). The mixture was allowed to stir at room temperature for 2 h. After completion of the reaction, 1 ml water was added and the resulted suspension was extracted with EtOAc. The organic layer was combined, dried over Na_2SO_4, filtered and concentrated under reduced pressure. The remaining residue was purified by silica gel (200-300 mesh) column chromatography with petroleum ehter : ethyl acetate : dichloromethane (20 : 2 : 5, v/v) as eluent to afford compound 1**8** (135 mg) as a white amorphous powder in 65% yield.

3-(3,5-Dimethoxyphenyl)-7-iodobenzofuran-5-carbaldehyde (**18**): white white amorphous powder. mp 178.9–181.7 °C; ^{1}H NMR (500 MHz, acetone-d$_6$): δ 10.11 (s, 1H), 8.52 (s, 1H), 8.42 (s, 1H), 8.33 (s, 1H), 6.90 (s, 2H), 6.59 (s, 1H), 3.87 (s, 6H). ^{13}C NMR (125 MHz, acetone-d$_6$): δ 191.41, 162.58 (2 × C), 159.81, 145.36, 135.51, 134.67, 133.36, 127.55, 125.24, 124.78, 106.57 (2 × C), 100.72, 77.10, 55.82 (2 × C). (+)-HRESI-MS: *m/z* 408.9926 [M+H]$^+$ (Calcd for $C_{17}H_{14}O_4I$: 408.9931).

3-(3,5-Dimethoxyphenyl)-2-(4-methoxyphenyl)benzofuran-5-carbaldehyde (19)

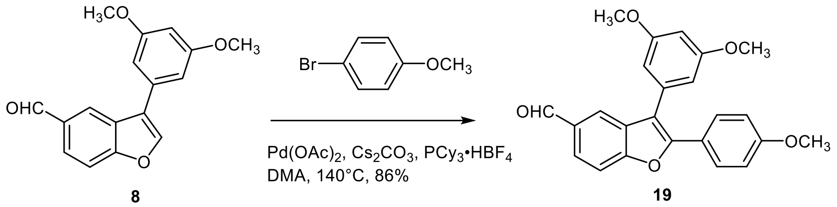

To a stirred solution of compound **8** (10.0 g, 35.45 mmol) in DMA (100 mL), Cs_2CO_3 (462 mg, 1.42 mmol), $Pd(OAc)_2$ (796 mg, 3.55 mmol), $PCy_3{\cdot}HBF_4$ (1044 mg, 2.84 mmol), and 4-bromoanisole (9888 mg, 35.45 mmol) were added in sequence at room temperature. The mixture was stirred in a sealed tube filled with dry argon at 140 °C for 18 h. After completion, the reaction mixture was filtered through celite and extracted with ethyl acetate. The organic layer was concentrated under reduced pressure, and the residue was recrystallized in acetone to provide compound **19** (11.8 g) as a light yellow oil in 80% yield.

3-(3,5-Dimethoxyphenyl)-2-(4-methoxyphenyl)benzofuran-5-carbaldehyde (**19**): light yellow oil. ^{1}H NMR (500 MHz, $CDCl_3$) δ 10.03 (s, 1H), 8.02 (d, J = 1.6 Hz, 1H), 7.87 (dd, J = 8.4, 1.7 Hz, 1H), 7.66 (d, J = 8.9 Hz, 2H), 7.63 (d, J = 8.5 Hz, 1H), 6.88 (d, J = 8.9 Hz, 2H), 6.63 (d, J = 2.3 Hz, 2H), 6.55 (t, J = 2.3 Hz, 1H), 3.83 (s, 3H), 3.80 (s, 6H). ^{13}C NMR (125 MHz, $CDCl_3$): δ 191.93, 161.54 (2 × C), 160.35, 157.30, 152.72, 134.05, 132.51, 131.30, 128.76 (2 × C), 125.95, 123.31, 122.47, 116.30, 114.18 (2 × C), 111.81, 107.83, 107.77, 100.24, 55.64 (2 × C), 55.58. (+)-HRESI-MS: *m/z* 389.1387 [M+H]$^+$ (Calcd for $C_{24}H_{21}O_5$: 389.1384).

(*E*)-3-(3,5-Dimethoxyphenyl)-5-(3,5-dimethoxystyryl)-2-(4-methoxyphenyl) benzofuran (20)

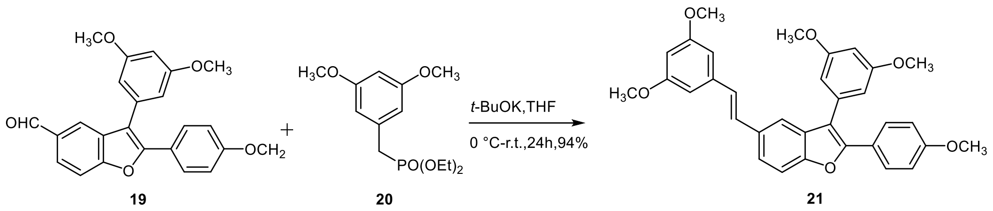

To a stirred solution of diethyl 3,5-dimethoxybenzylphosphonate **20** (7.4 g, 25.70 mmol) in freshly redistilled THF (300 ml) was added 2.9 g *t*-BuOK (25.70 mmol) at –40 °C. After stirring for 20 min, a solution of compound **19** (5.0 g, 12.85 mmol) in freshly redistilled THF (200 ml) was added. The resulted mixture was allowed to warm to room temperature and stirred for another 24 h. After removing of the solvent under reduced pressure, the residue was redissolved in ethyl acetate. The organic layer was washed successively with brine, water, dried over anhydrous Na_2SO_4, and concentrated in vacuo. The remaining residue was then purified by silica gel (200-300 mesh) column chromatography with petroleum and acetone (15 : 1, v/v) as eluent to provide compound **21** (6.3 g) as a colorless oil in 94% yield.

(*E*)-3-(3,5-Dimethoxyphenyl)-5-(3,5-dimethoxystyryl)-2-(4-methoxyphenyl) benzofuran (**21**): colorless oil. ^{1}H NMR (500 MHz, $CDCl_3$) δ 7.64 (d, J = 8.8 Hz, 2H), 7.58 (s, 1H), 7.50 (m, 2H), 7.17 (d, J = 16.2 Hz, 1H), 7.00 (d, J = 16.2 Hz, 2H), 6.87 (d, J = 8.9 Hz, 2H), 6.67 (m, 4H), 6.54 (t, J = 2.3 Hz, 1H), 6.39 (t, J = 2.2 Hz, 1H), 3.83 (s, 6H), 3.83 (s, 3H), 3.80 (s, 6H). ^{13}C NMR (125 MHz, $CDCl_3$) δ 161.41 (2 × C), 161.11 (2 × C), 159.97, 153.74, 151.54, 139.74, 135.03, 132.57, 130.99, 129.68, 128.64, 128.61, 127.69, 123.23, 123.19, 118.03, 117.99, 116.10, 114.06, 111.27, 107.91, 107.86, 104.53, 104.48, 100.12, 100.08, 55.66 (2 × C), 55.60, 55.56, 55.50. (+)-HRESI-MS: *m/z* 523.2120 [M+H]$^+$ (Calcd for $C_{33}H_{31}O_6$: 523.2115).

(*E*)-5-(2-(3-(3,5-Dihydroxyphenyl)-2-(4-hydroxyphenyl)benzofuran-5-yl)vinyl) benzene-1,3-diol (1)

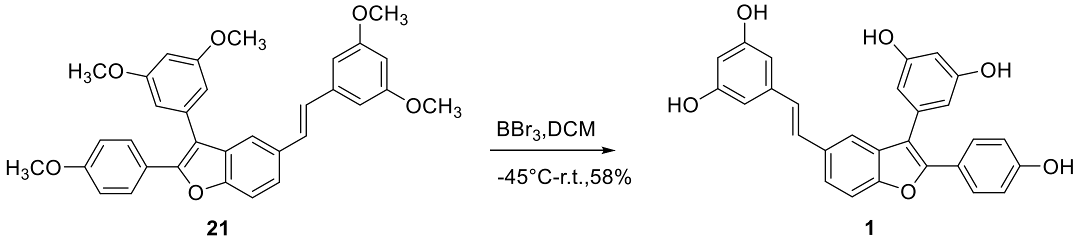

To a solution of compound **21** (6.0 g, 13.25 mmol) in dichloromethane (500 ml), a solution of BBr_3 (138 ml, 1 mol/l) in freshly redistilled dichloromethane (100 ml) was added dropwise within 30 min at –45 °C. The mixture was stirred for 2 h at the same

temperature under argon atmosphere. Then the mixture was warmed up to –25 °C, –10 °C, 0 °C, and stirred for another 2 h, respectively. After stirred overnight at room temperature, methanol (150 ml) was added dropwise at –45 °C to quench the reaction. The mixture was diluted with 80 ml EtOAc, washed with water, dried over anhydrous Na_2SO_4, and evaporated under reduced pressure. The remaining residue was subsequently separated by silica gel (200-300 mesh) column chromatography (dichloromethane: methanol = 40: 1, v/v) to give compound **1** as a white amorphous powder (3.5 g, 58%).

(*E*)-5-(2-(3-(3,5-Dihydroxyphenyl)-2-(4-hydroxyphenyl)benzofuran-5-yl)vinyl) benzene-1,3-diol (**1**): white amorphous powder. ^{1}H NMR (500 MHz, CD_3OD) δ 7.56 (d, J = 1.6 Hz, 1H), 7.54 (d, J = 8.8 Hz, 2H), 7.52-7.49 (m, 1H), 7.49 (d, J = 8.5 Hz, 1H), 7.13 (d, J = 16.2 Hz, 1H), 6.95 (d, J = 16.2 Hz, 1H), 6.78 (d, J = 8.7 Hz, 2H), 6.49 (d, J = 2.2 Hz, 2H), 6.43 (d, J = 2.2 Hz, 2H), 6.35 (t, J = 2.2 Hz, 1H), 6.18 (t, J = 2.2 Hz, 1H). ^{13}C NMR (125 MHz, CD_3OD): δ 160.32 (2 × C), 159.74 (2 × C), 159.56, 154.83, 152.91, 141.00, 136.02, 134.07, 132.09, 129.81, 129.66 (2 × C), 128.79, 123.90, 123.01, 118.55, 116.78, 116.41 (2 × C), 111.84, 109.15 (2 × C), 105.95 (2 × C), 103.04, 102.98. (+)-HRESI-MS: *m/z* 453.1336 $[M+H]^+$ (Calcd for $C_{28}H_{21}O_6$: 453.1333).[1]

Notes and references

1. D. D. Voa and M. Elofsson, *Adv. Synth. Catal.*, 2016, **358**, 4085.

Section 3. Spectra in the total synthesis of dehydro-δ-viniferin (1)

3.1 Spectra of 11

^{1}H NMR of Compound **11**

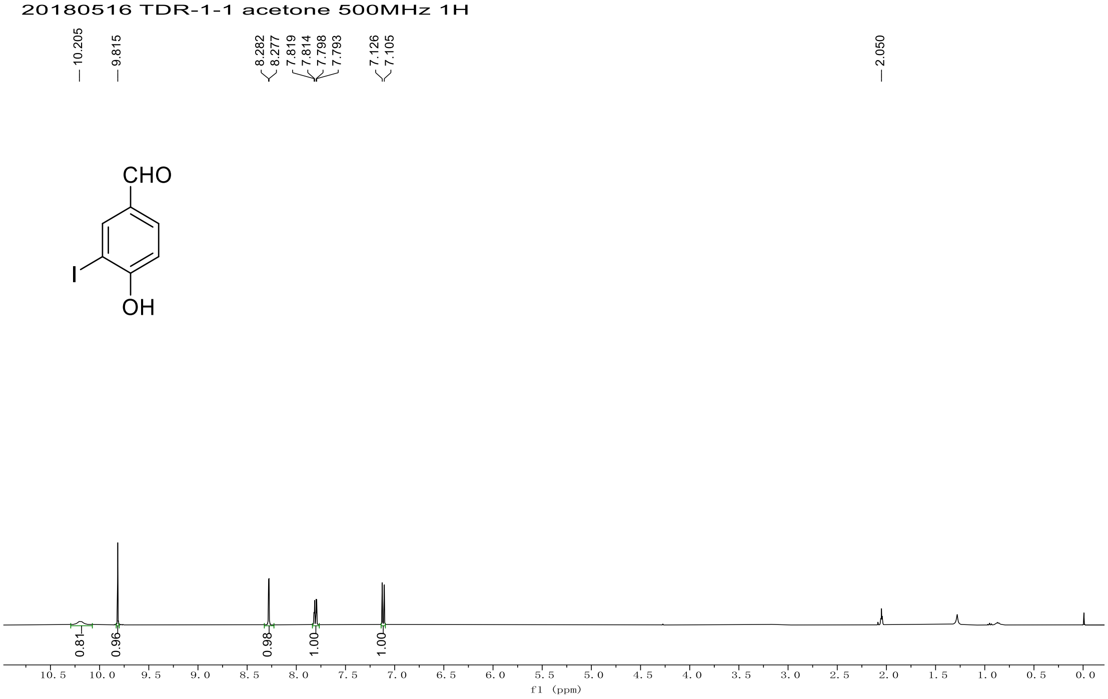

^{13}C NMR of Compound **11**

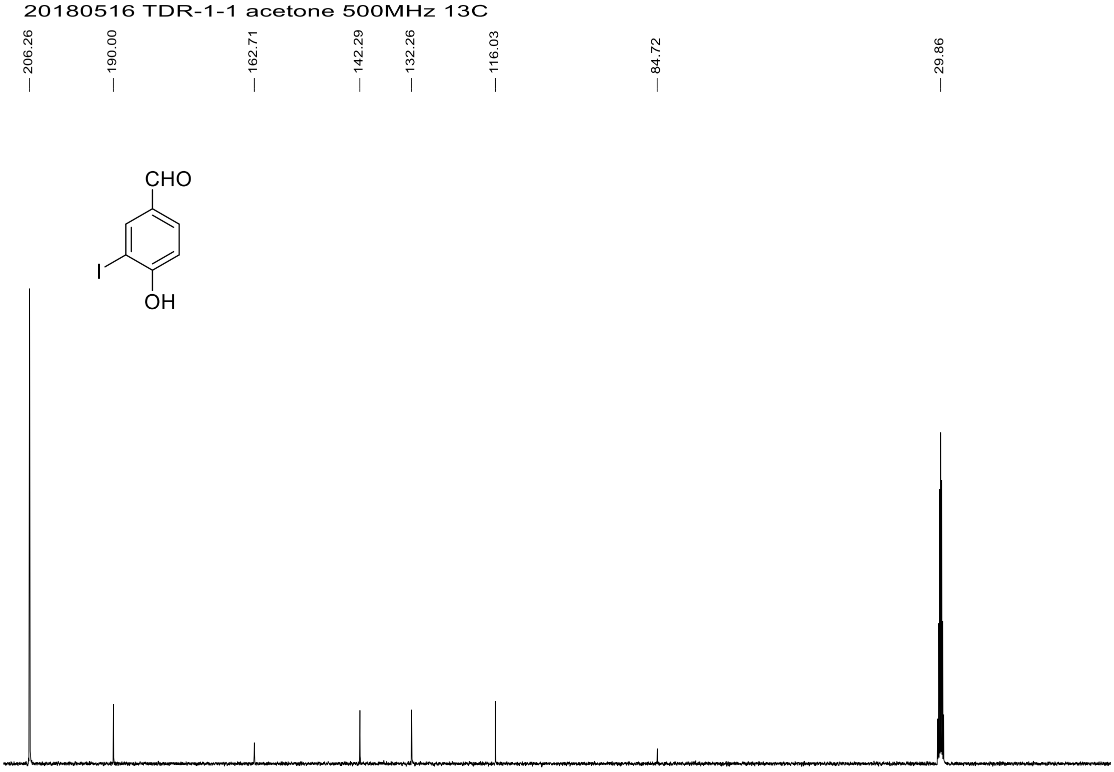

HRESI of Compound **11**

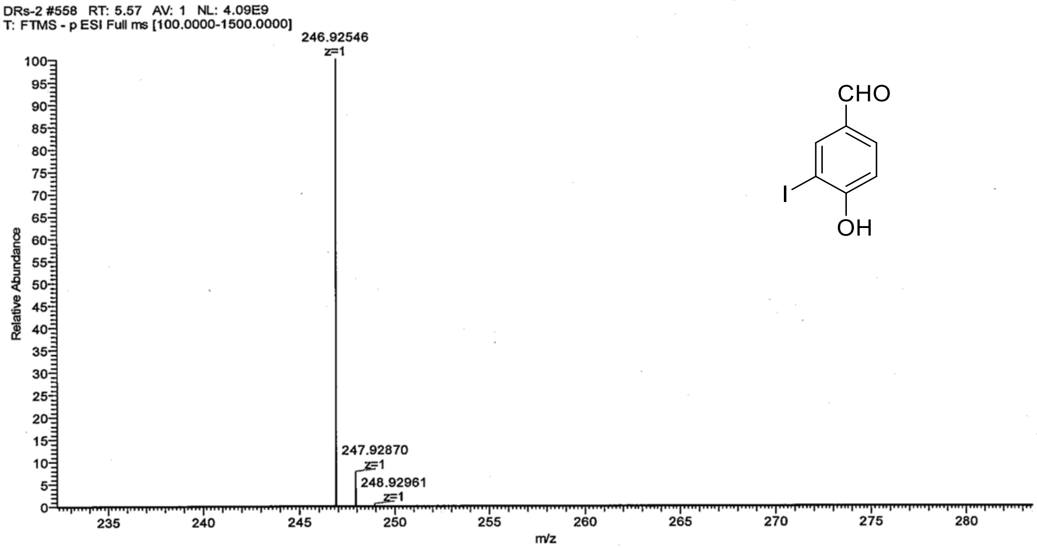

m/z	Theo. Mass	Delta (ppm)	RDB equiv.	Composition	
246.92546	246.92615	-2.78	5.5	C7 H4 O2 I	M-H

3.2 Spectra of 12

^{1}H NMR of Compound **12**

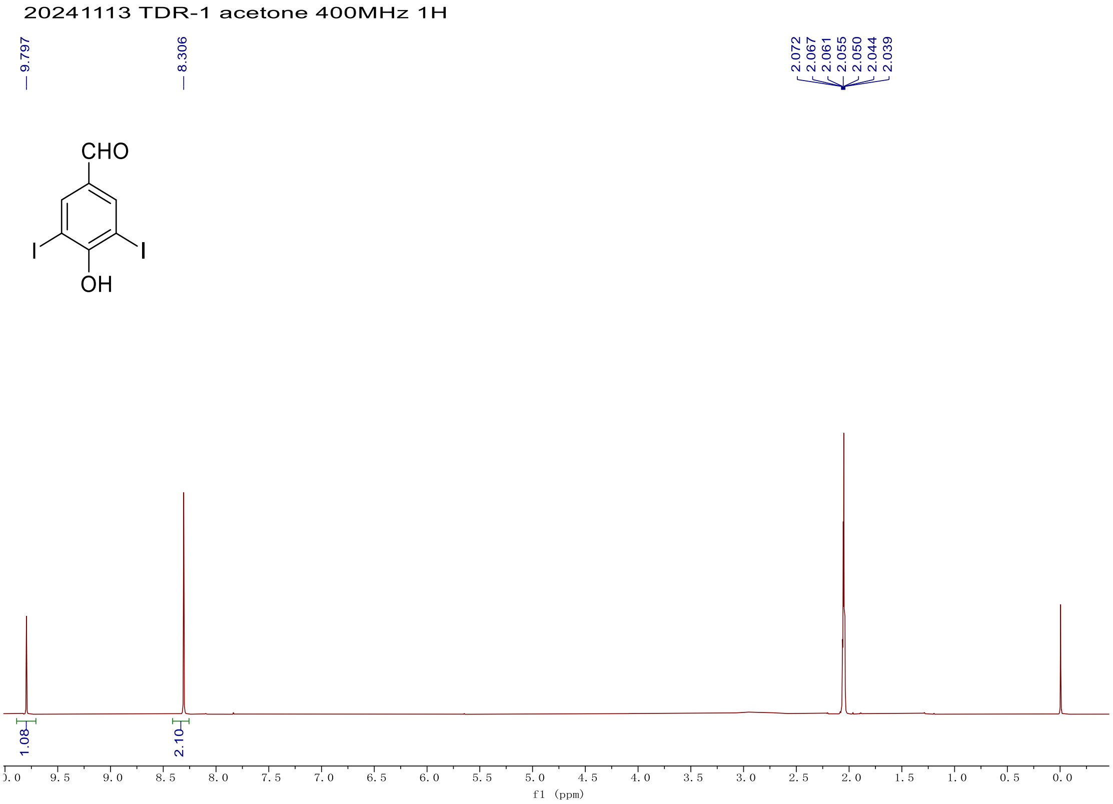

^{13}C NMR of Compound **12**

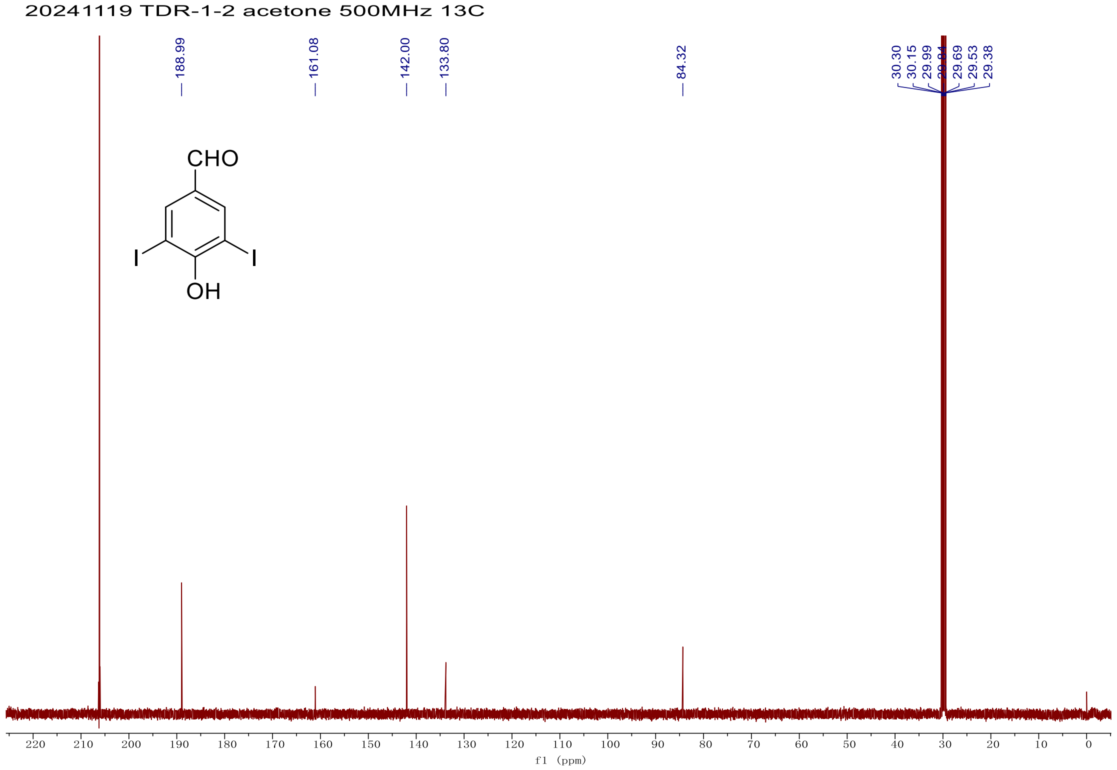

HRESI of Compound **12**

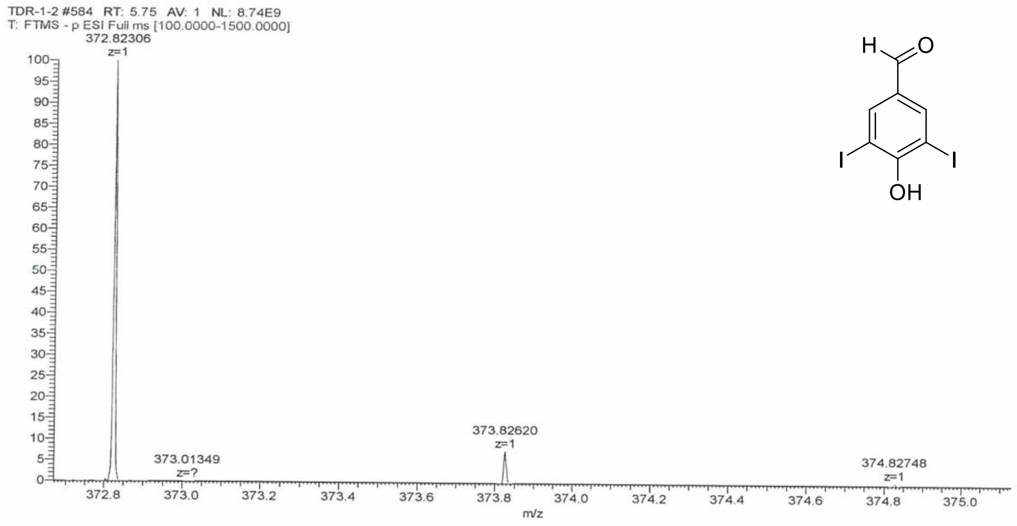

m/z	Theo. Mass	Delta (ppm)	RDB equiv.	Composition	
372.82306	372.82279	0.73	5.5	C7 H3 O2 I2	M-H

3.3 Spectra of 13

^{1}H NMR of Compound **13**

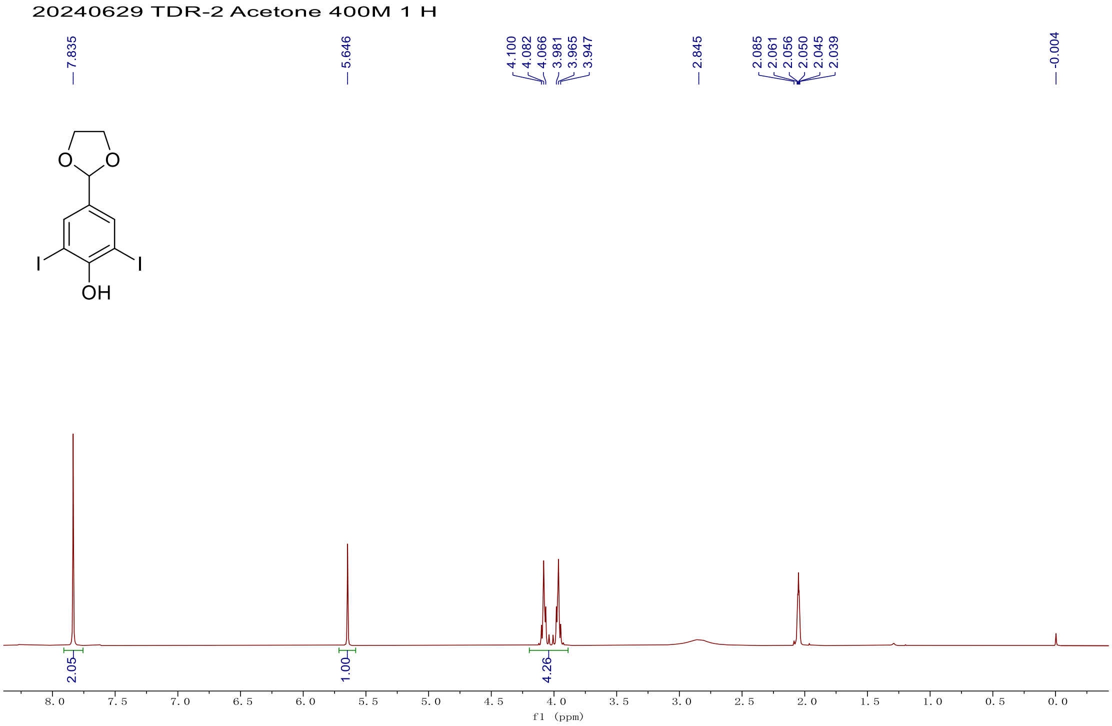

^{13}C NMR of Compound **13**

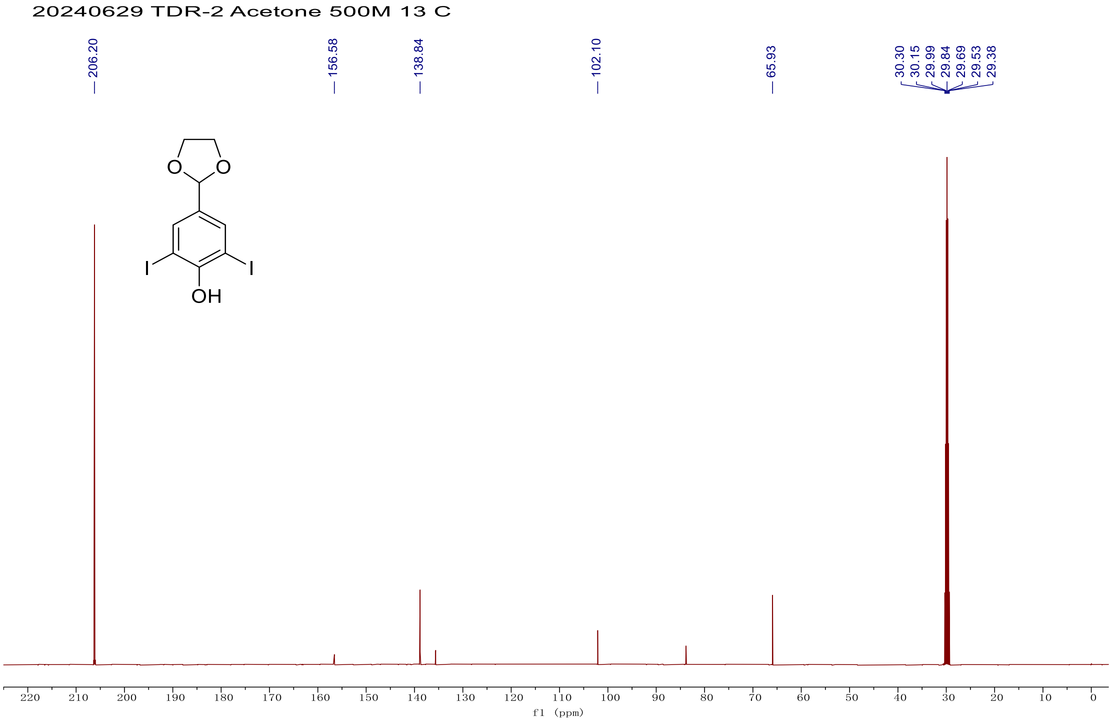

HRESI of Compound **13**

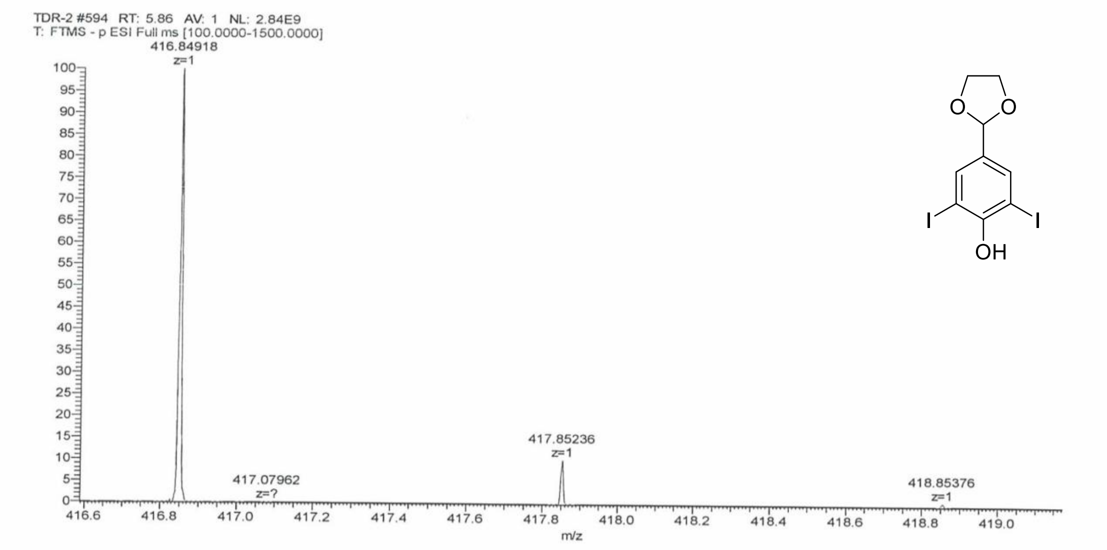

m/z	Theo. Mass	Delta (ppm)	RDB equiv.	Composition	
416.84918	416.84900	0.42	5.5	C9 H7 O3 I2	M-H

3.4 Spectra of 10a

^{1}H NMR of Compound **10a**

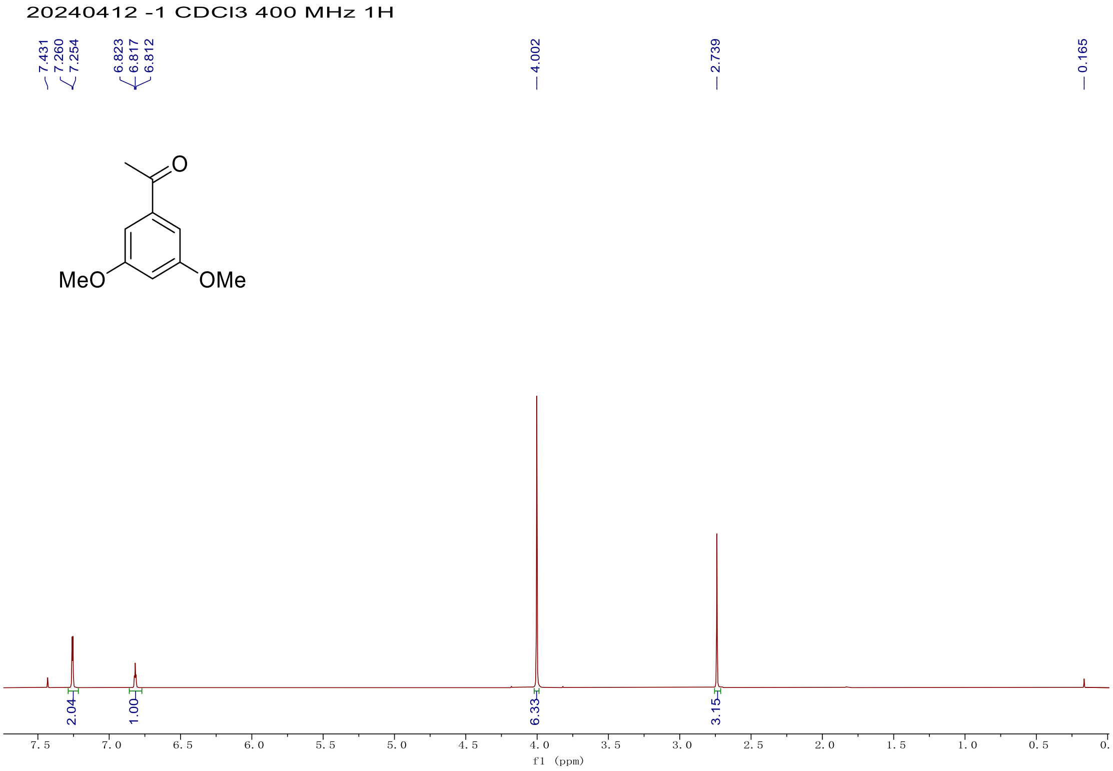

^{13}C NMR of Compound **10a**

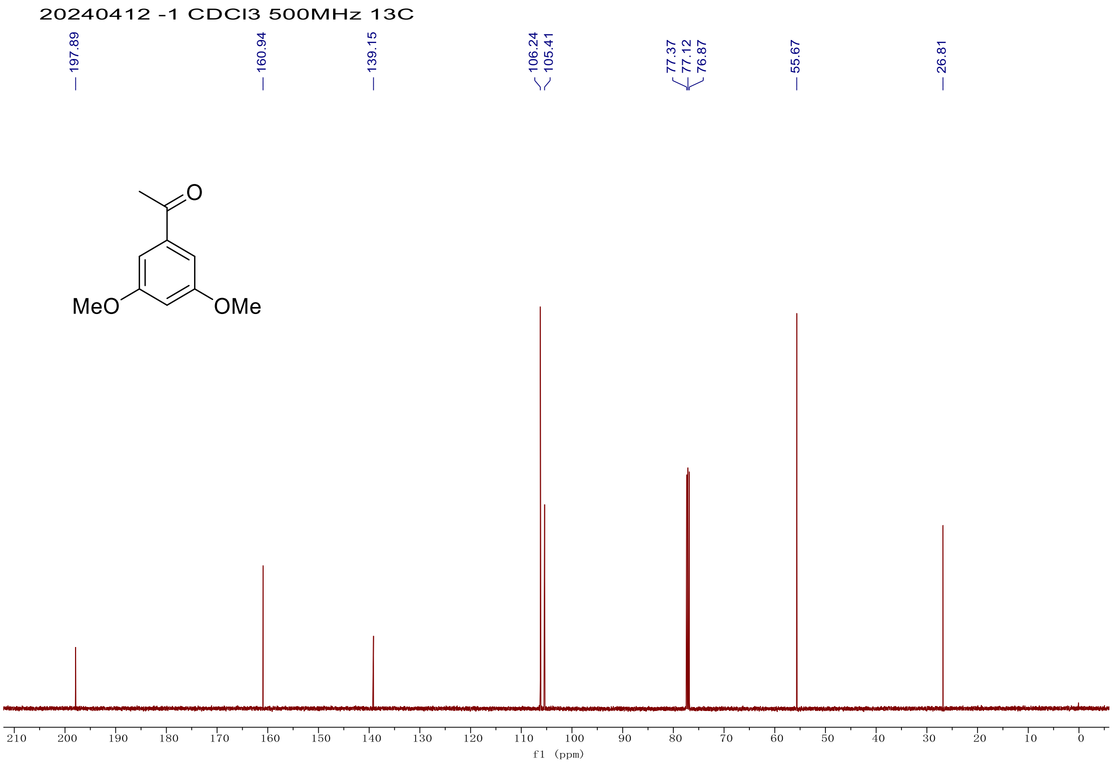

3.5 Spectra of 15

^{1}H NMR of Compound **15**

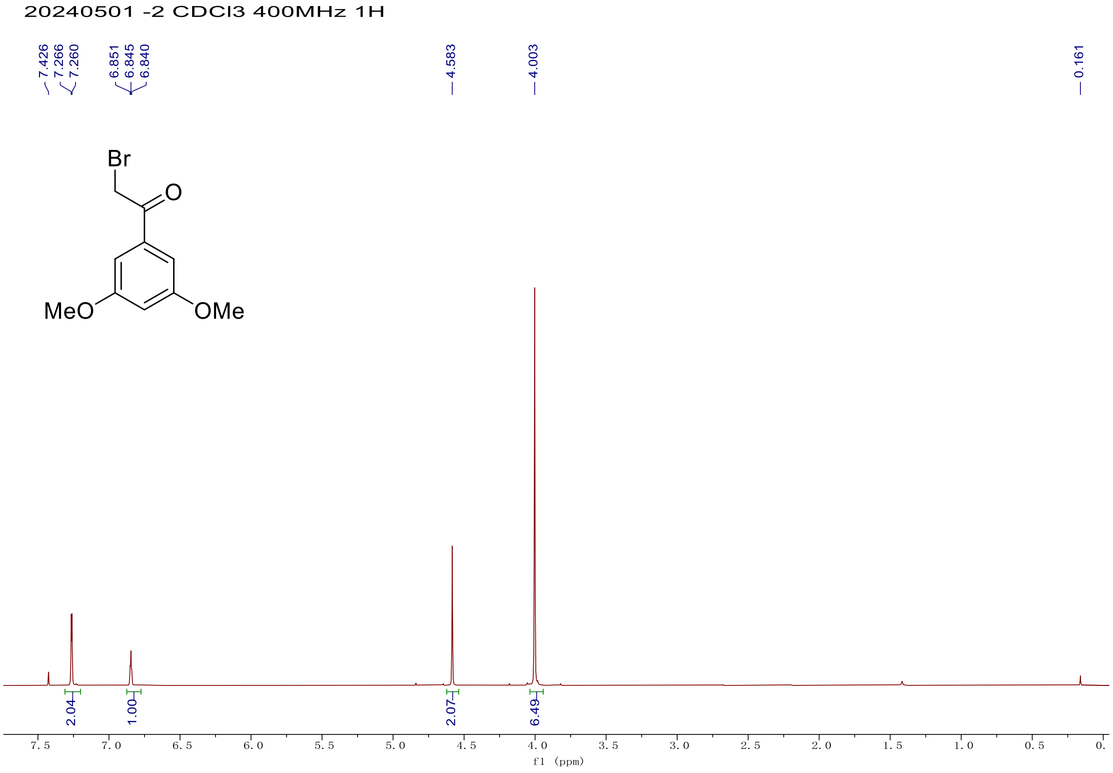

^{13}C NMR of Compound **15**

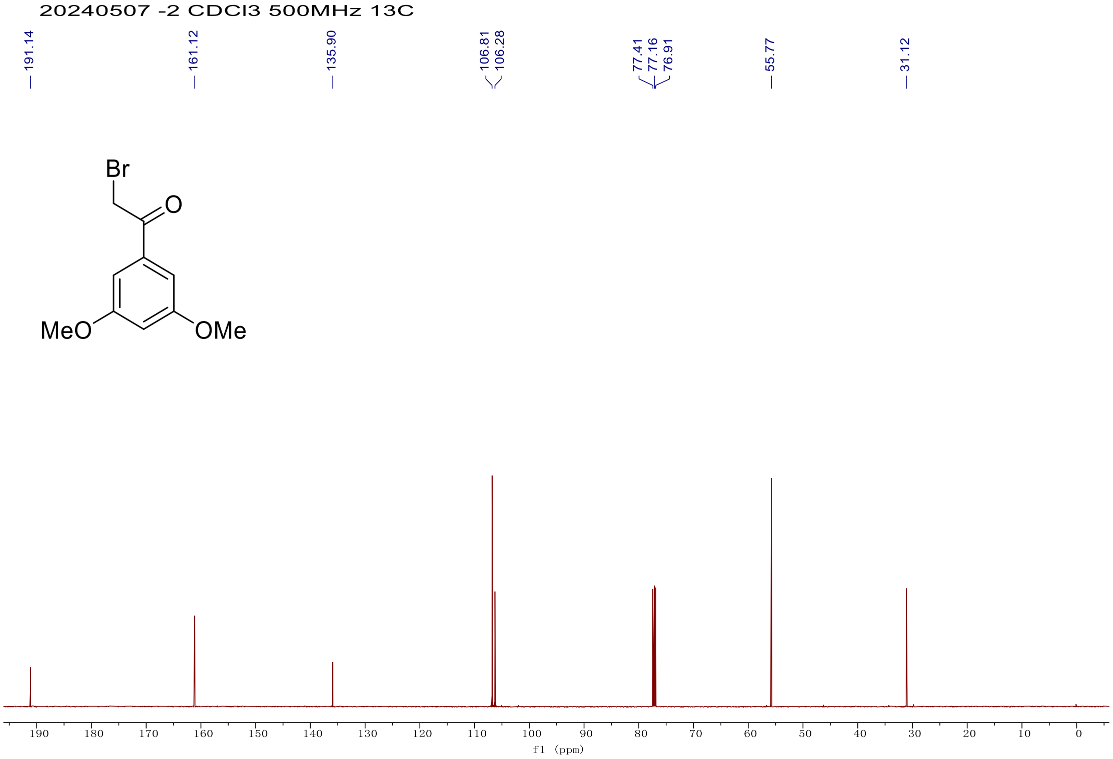

ESI of Compound **15**

Single Mass Spectrum Deconvolution Report

Analysis Name: zhgjf005.d **Instrument:** LC-MSD-Trap-SL **Print Date:** 6/27/2014 10:58:44 AM
Method: TEST.MS **Operator:** Operator **Acq. Date:** 6/27/2014 10:11:52 AM
Sample Name: Svm-2
Analysis Info:

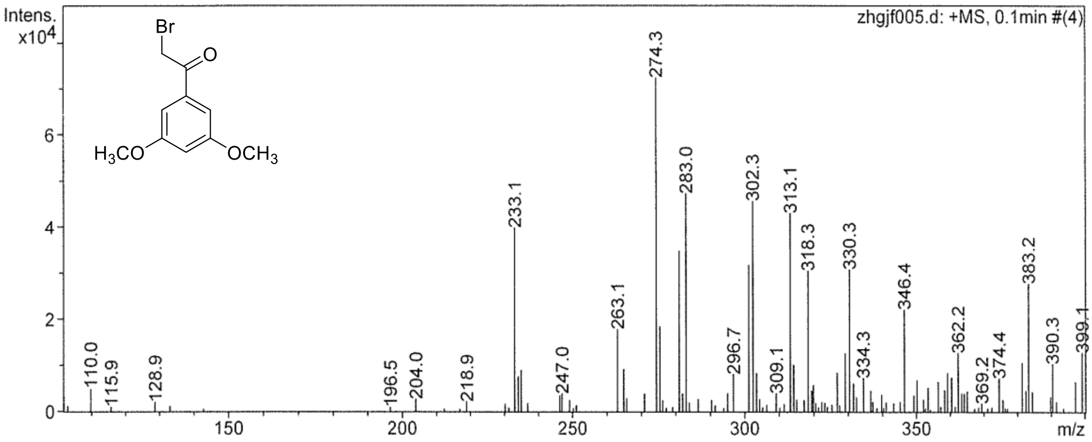

3.6 Spectra of 14

^{1}H NMR of Compound **14**

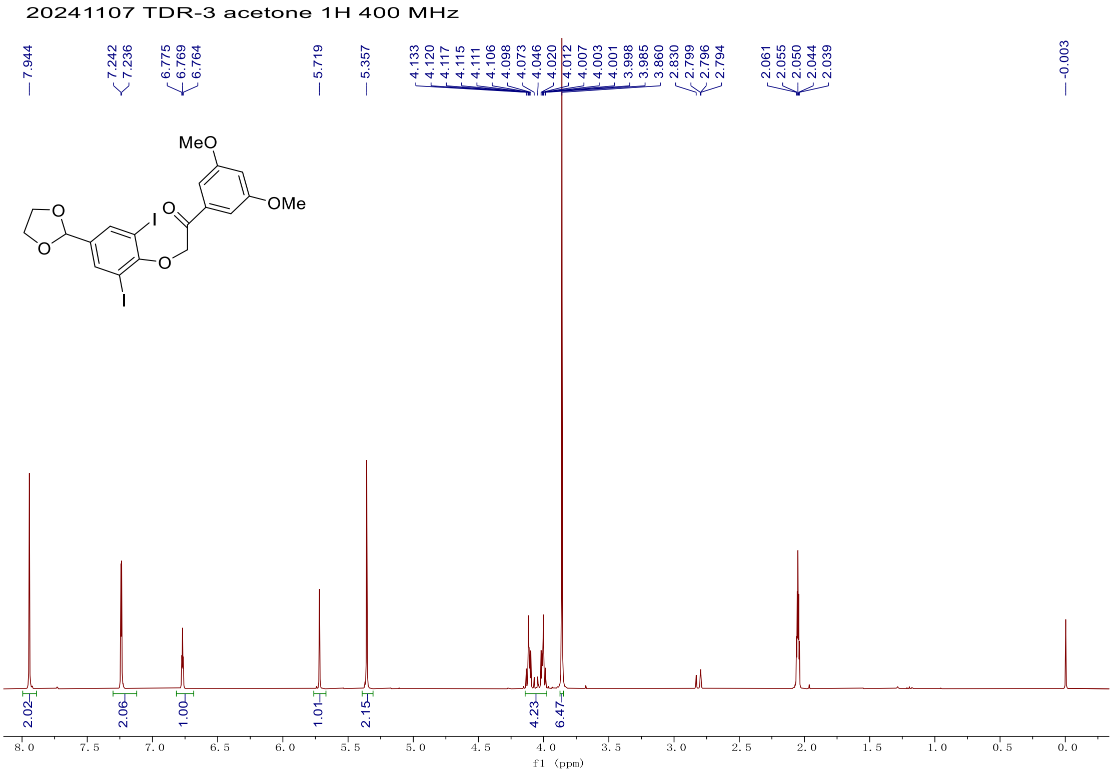

^{13}C NMR of Compound **14**

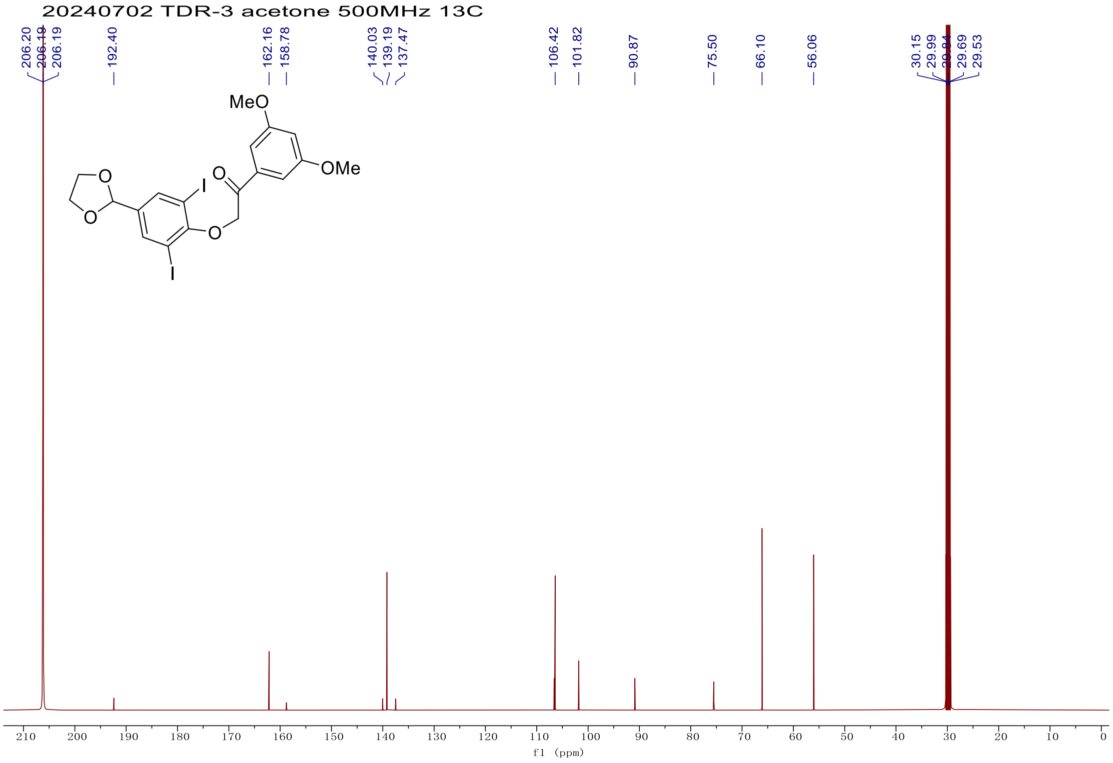

HRESI of Compound **14**

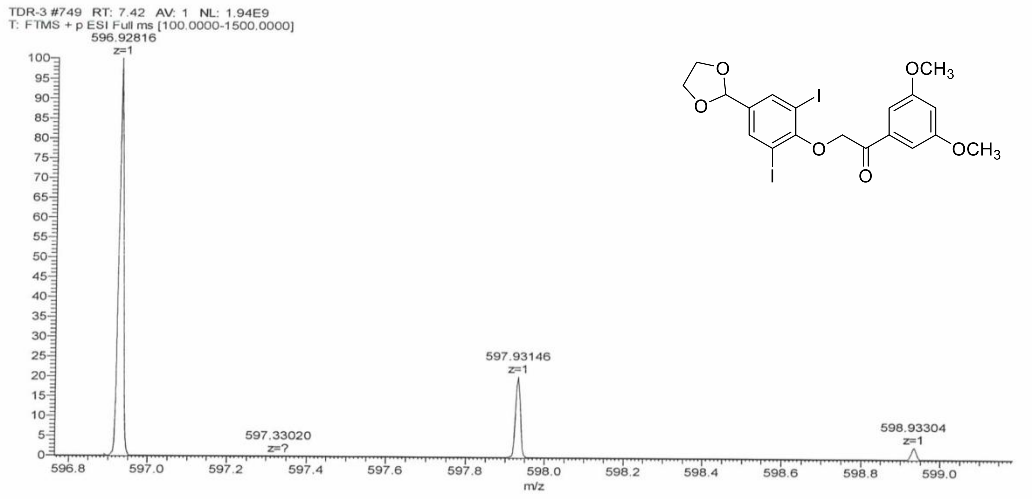

m/z	Theo. Mass	Delta (ppm)	RDB equiv.	Composition	
596.92816	596.92655	2.7	9.5	C19 H19 O6 I2	M+H

3.7 Spectra of 16

^{1}H NMR of Compound **16**

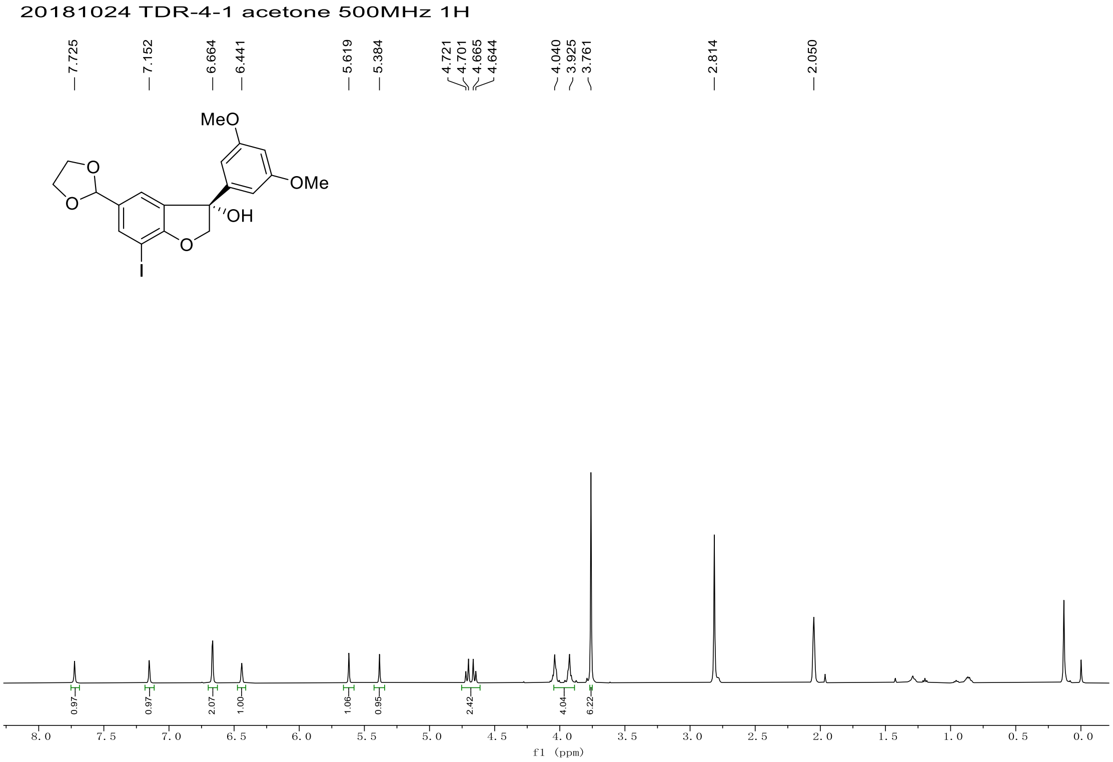

^{13}C NMR of Compound **16**

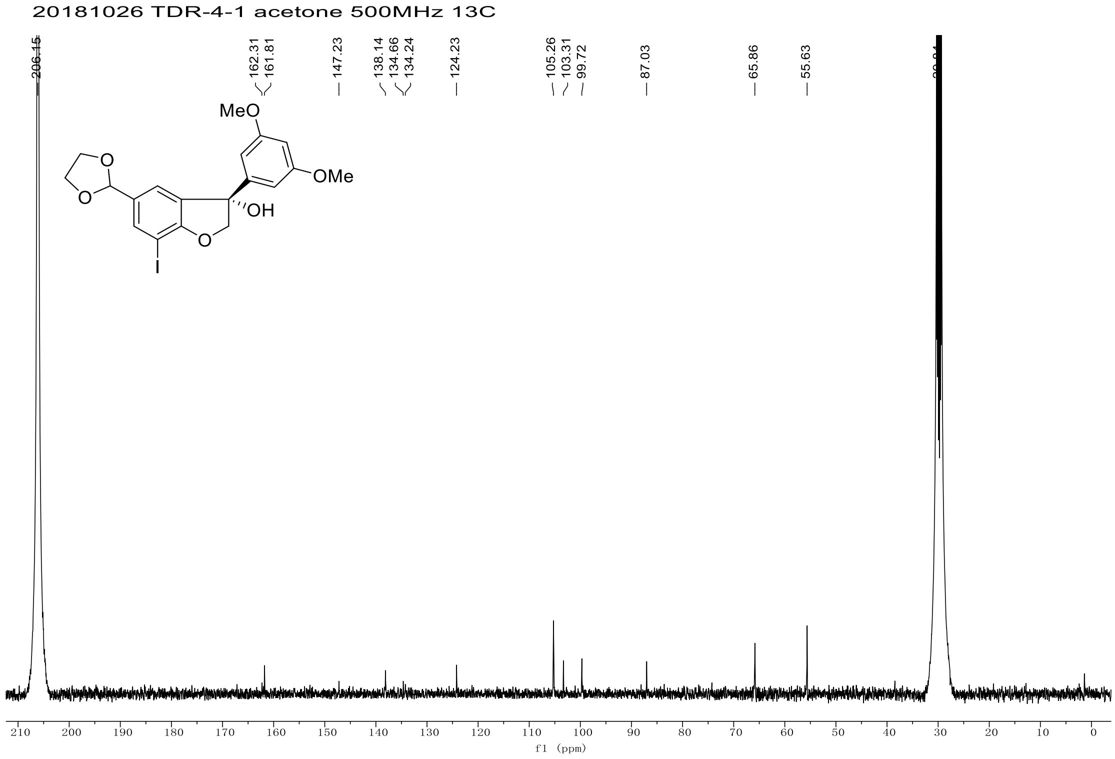

HRESI of Compound **16**

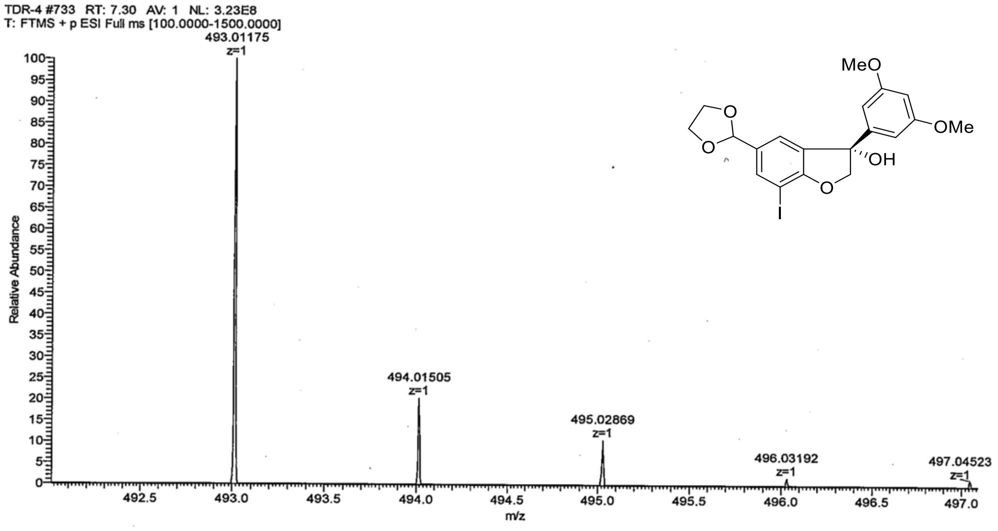

m/z	Theo. Mass	Delta (ppm)	RDB equiv.	Composition	
493.01175	493.01185	-0.21	9.5	C19 H19 O6 I Na	M+Na

3.8 Spectra of 17

^{1}H NMR of Compound **17**

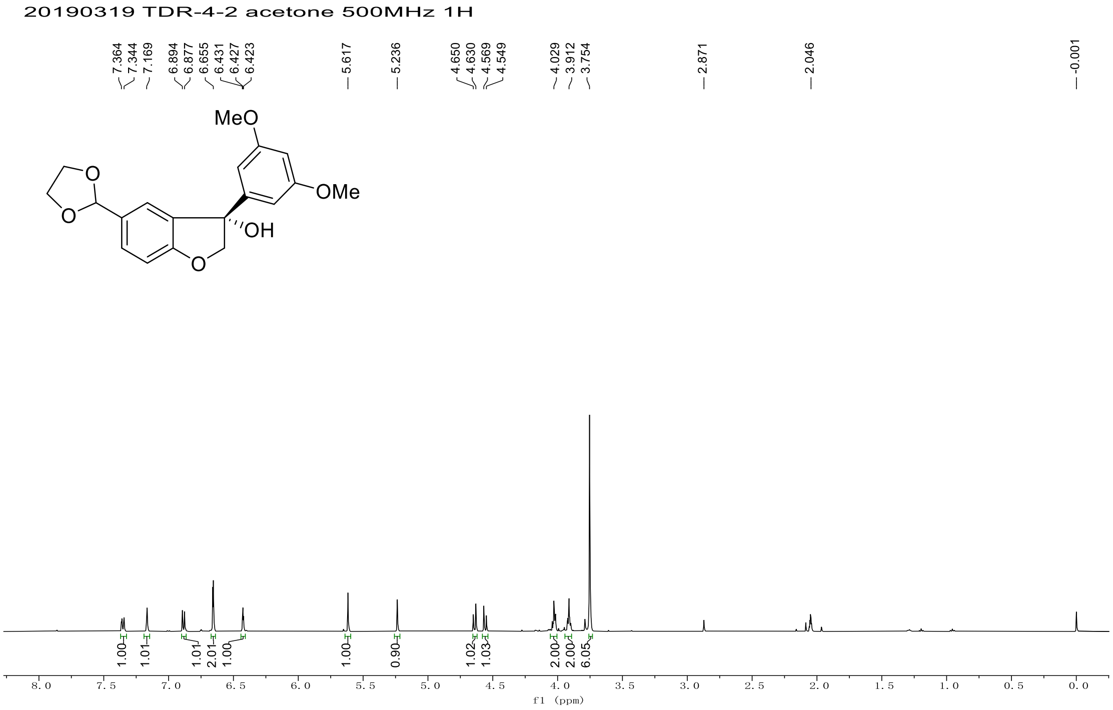

^{13}C NMR of Compound **17**

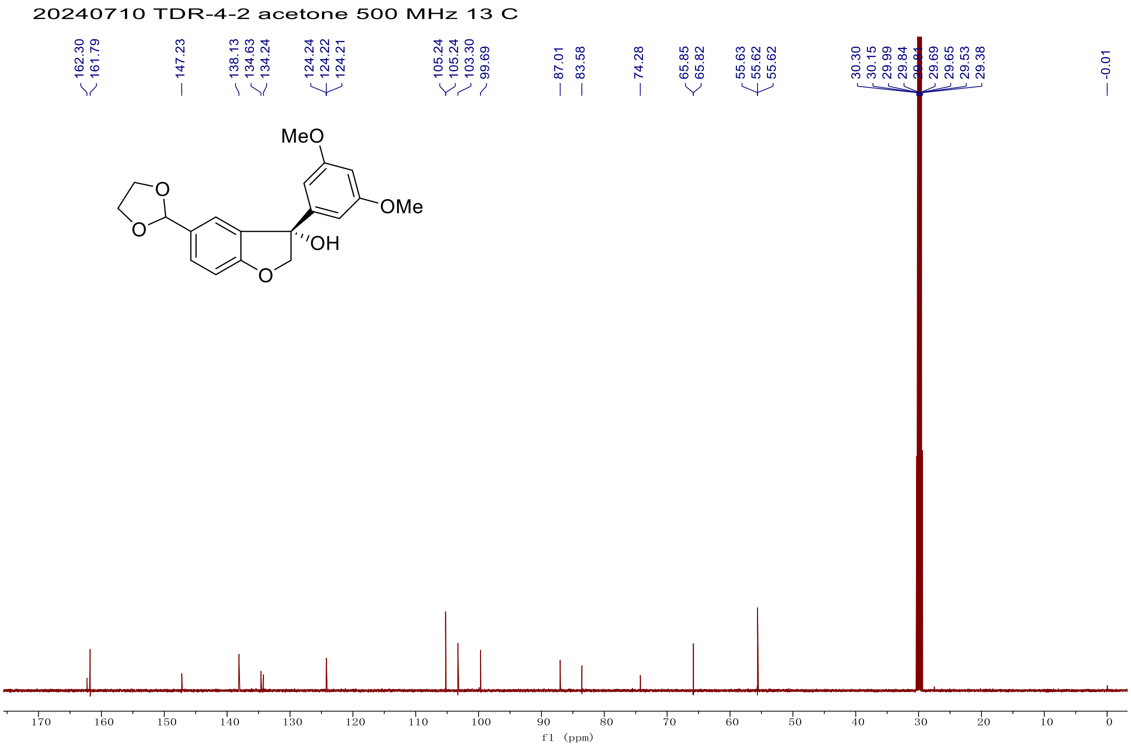

HRESI of Compound **17**

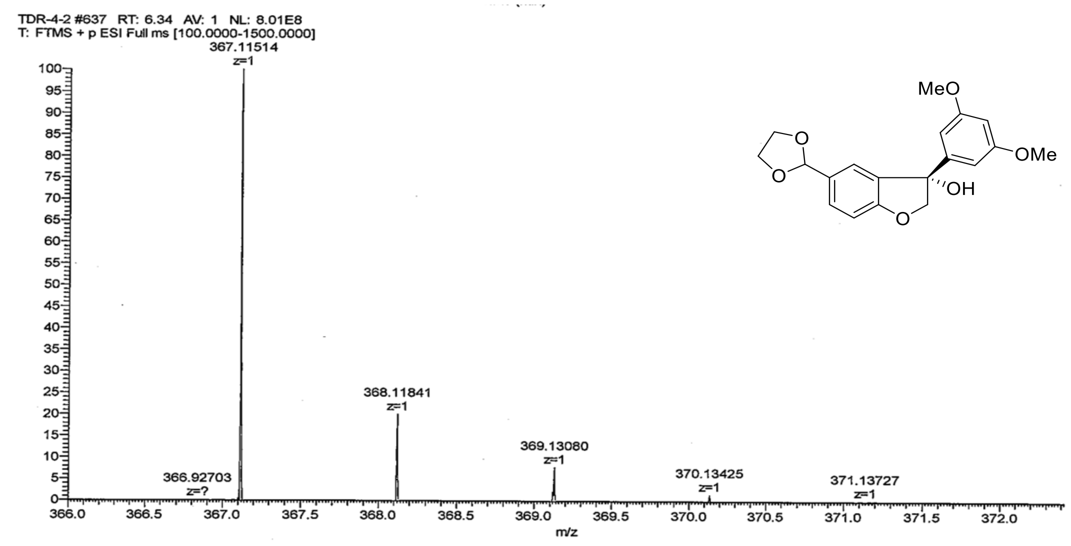

m/z	Theo. Mass	Delta (ppm)	RDB equiv.	Composition	
367.11514	367.11521	-0.19	9.5	C19 H20 O6 Na	M+Na

3.9 Spectra of 8

^{1}H NMR of Compound **8**

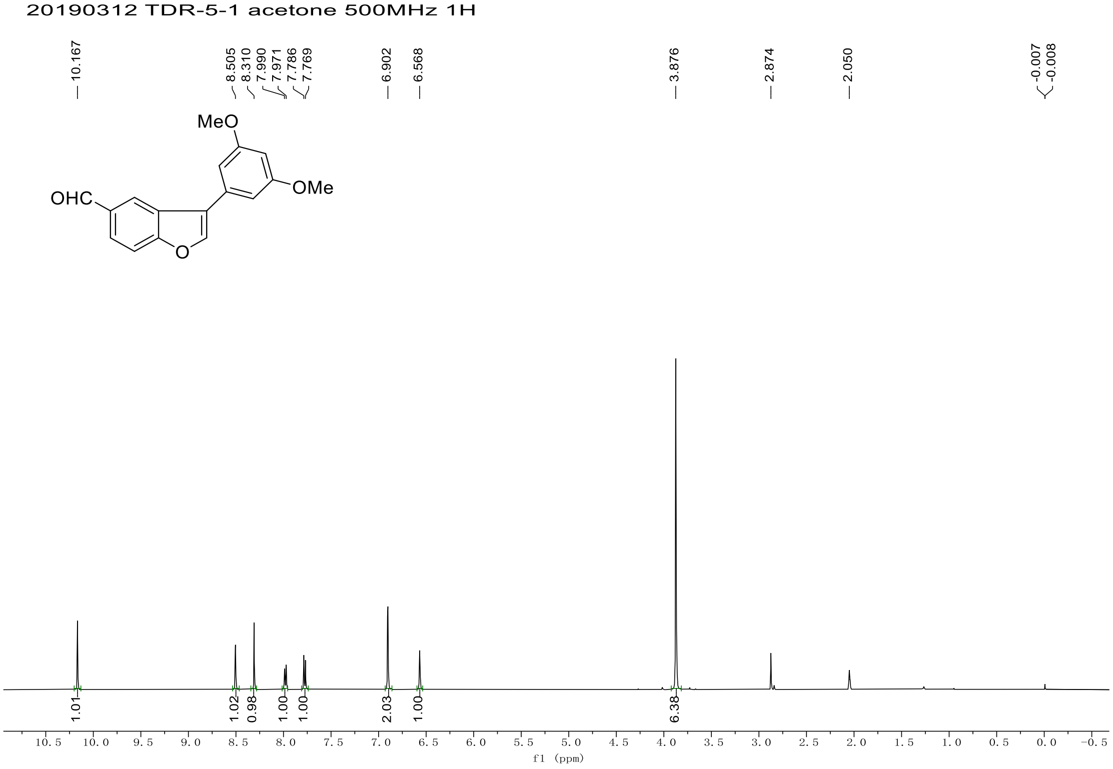

^{13}C NMR of Compound **8**

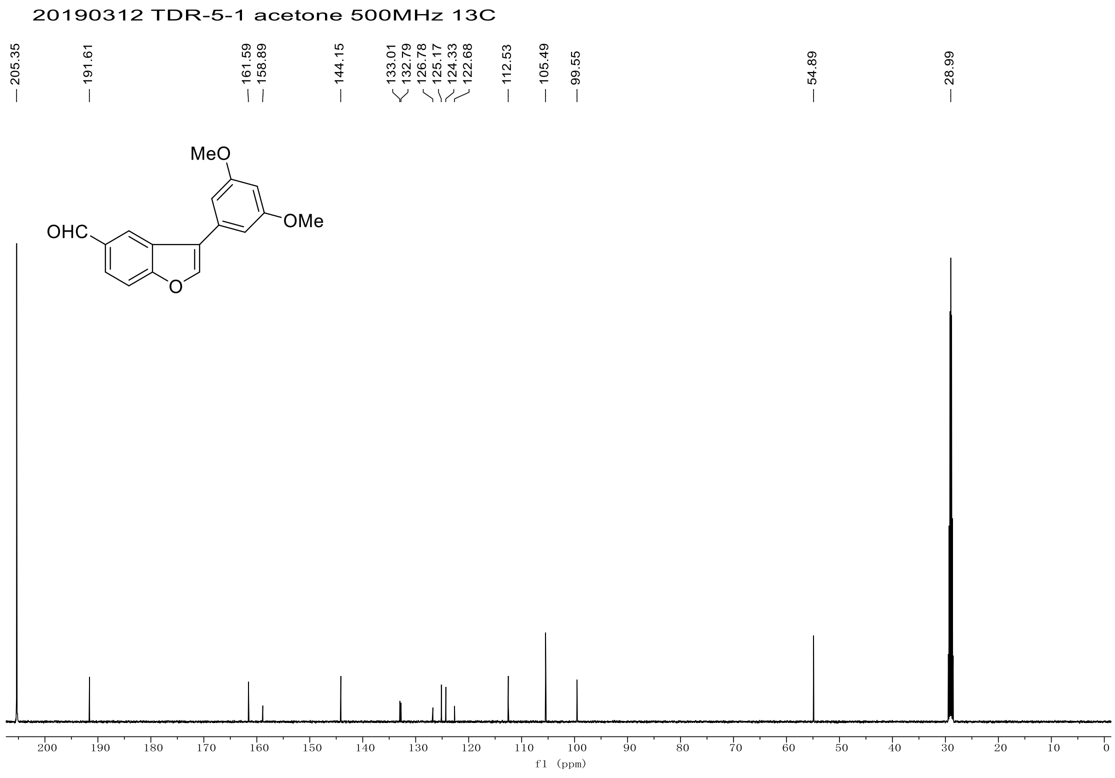

HRESI of Compound **8**

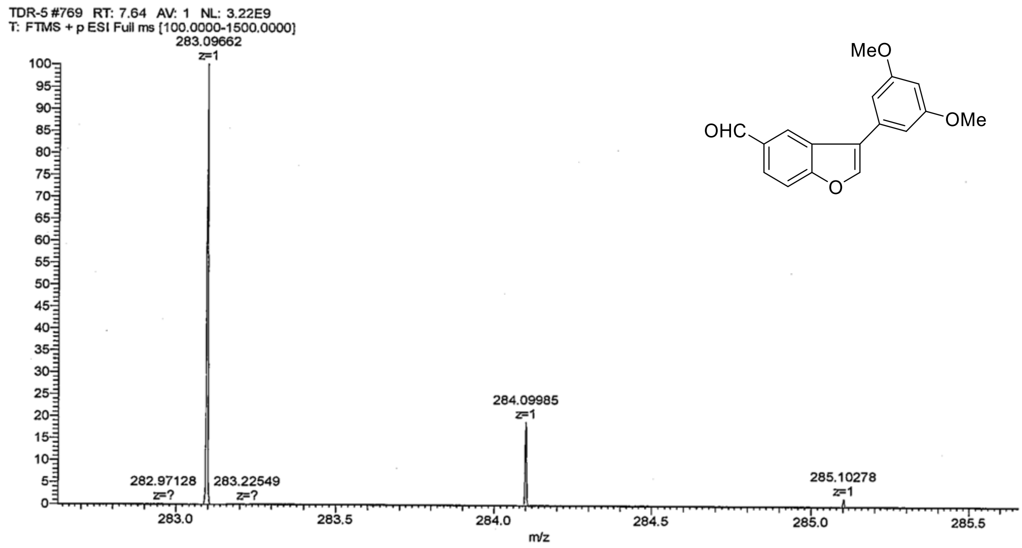

m/z	Theo. Mass	Delta (ppm)	RDB equiv.	Composition	
283.09662	283.09649	0.48	10.5	C17 H15 O4	M+H

3.10 Spectra of 18

^{1}H NMR of Compound **18**

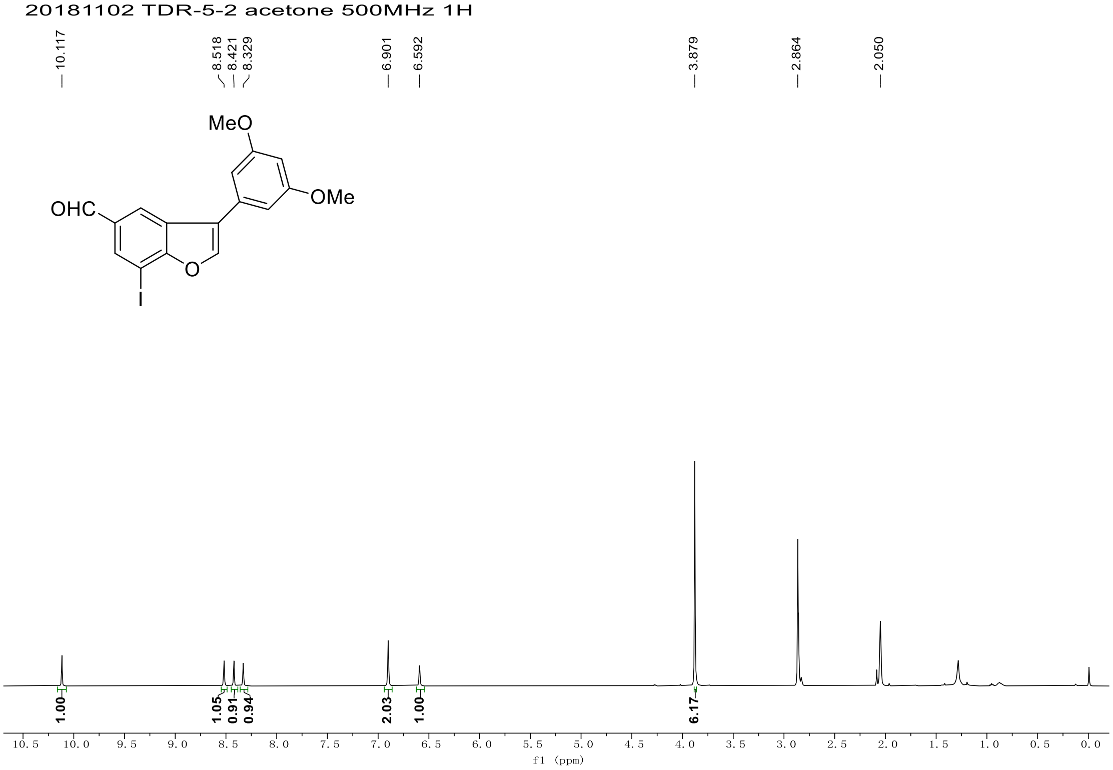

^{13}C NMR of Compound **18**

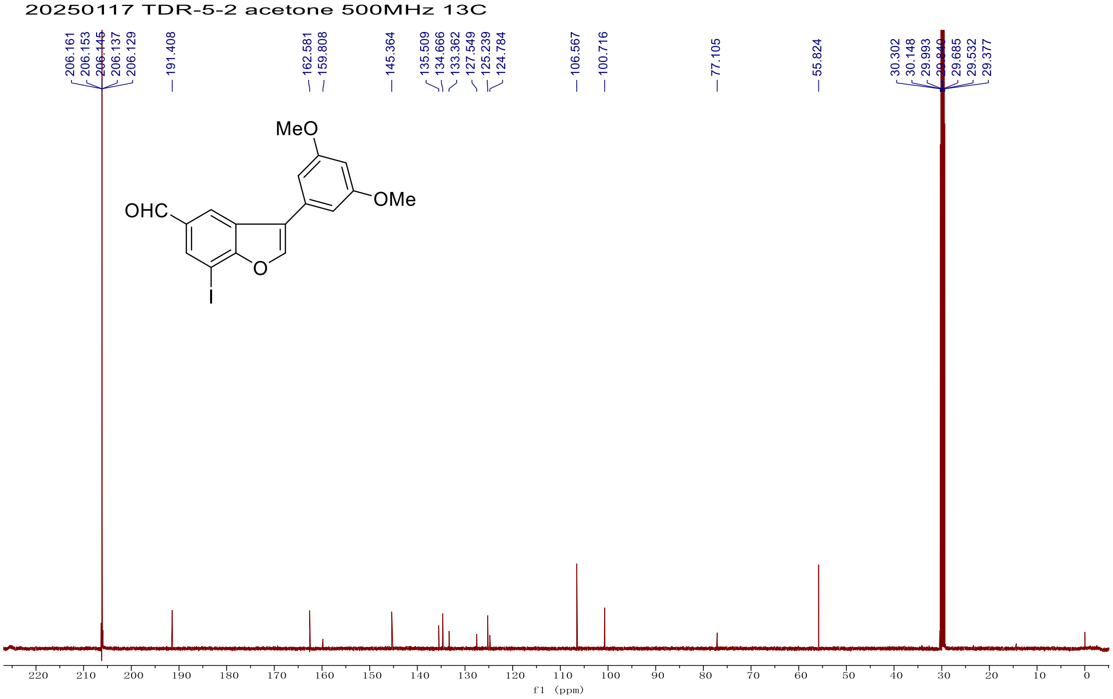

HRESI of Compound **18**

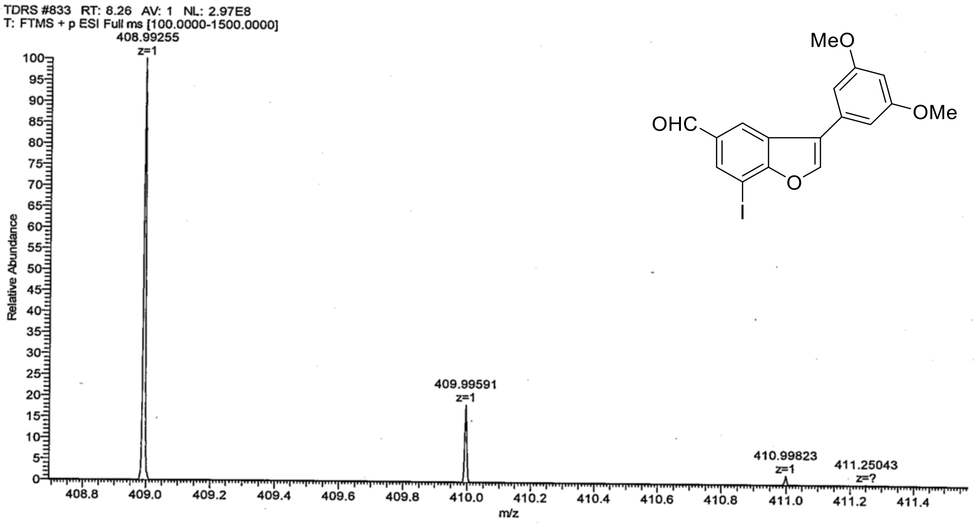

m/z	Theo. Mass	Delta (ppm)	RDB equiv.	Composition	
408.99255	408.99313	-1.41	10.5	C17 H14 O4 I	M+H

3.11 Spectra of 19

^{1}H NMR of Compound **19**

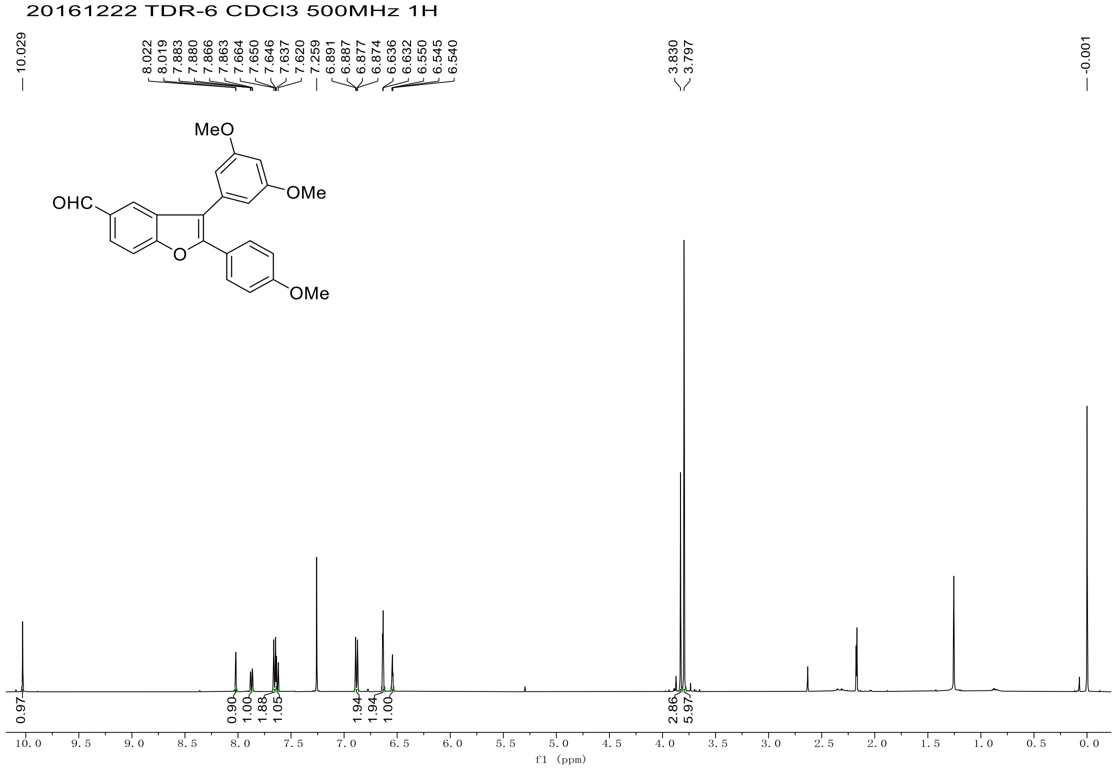

^{13}C NMR of Compound **19**

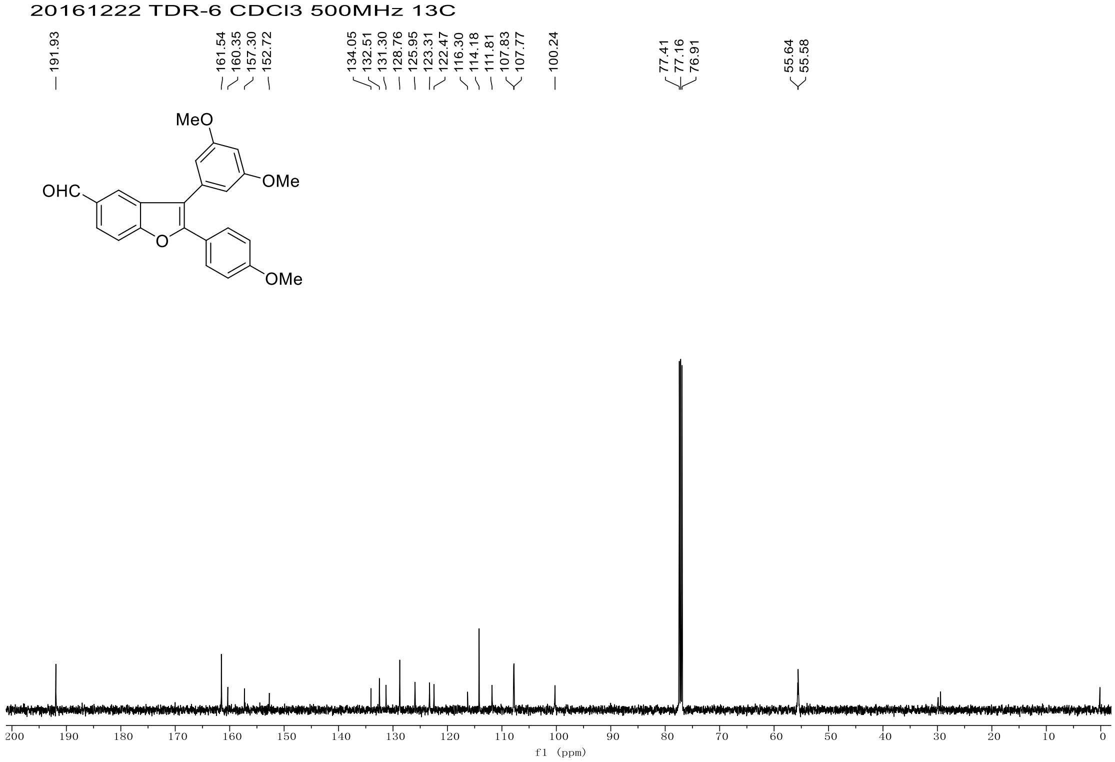

HRESI of Compound **19**

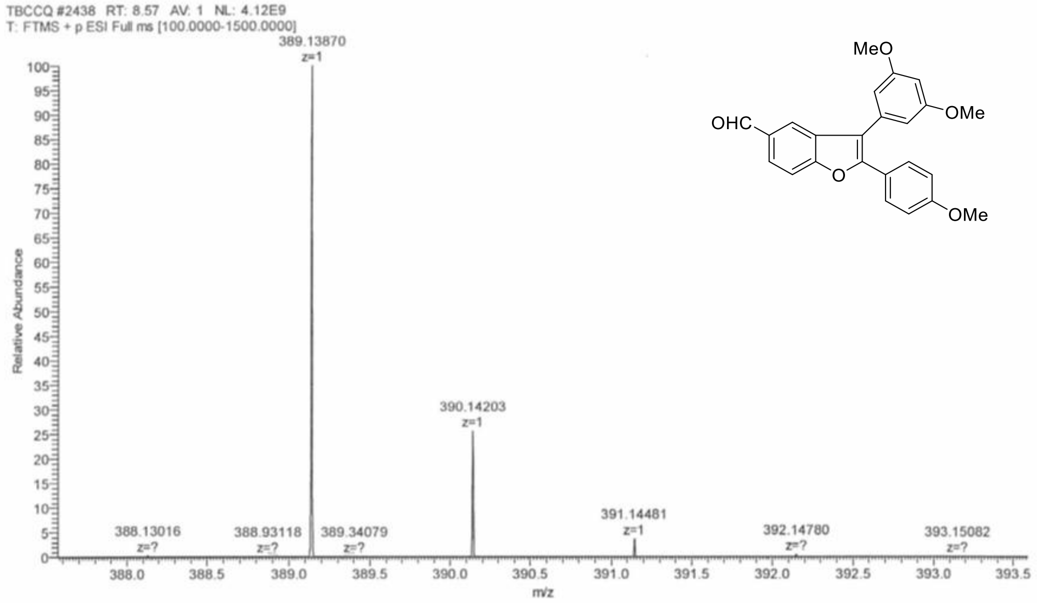

m/z	Theo. Mass	Delta (ppm)	RDB equiv.	Composition	
389.13870	389.13835	0.9	14.5	C24 H21 O5	M+H

3.12 Spectra of 20

^{1}H NMR of Compound **21**

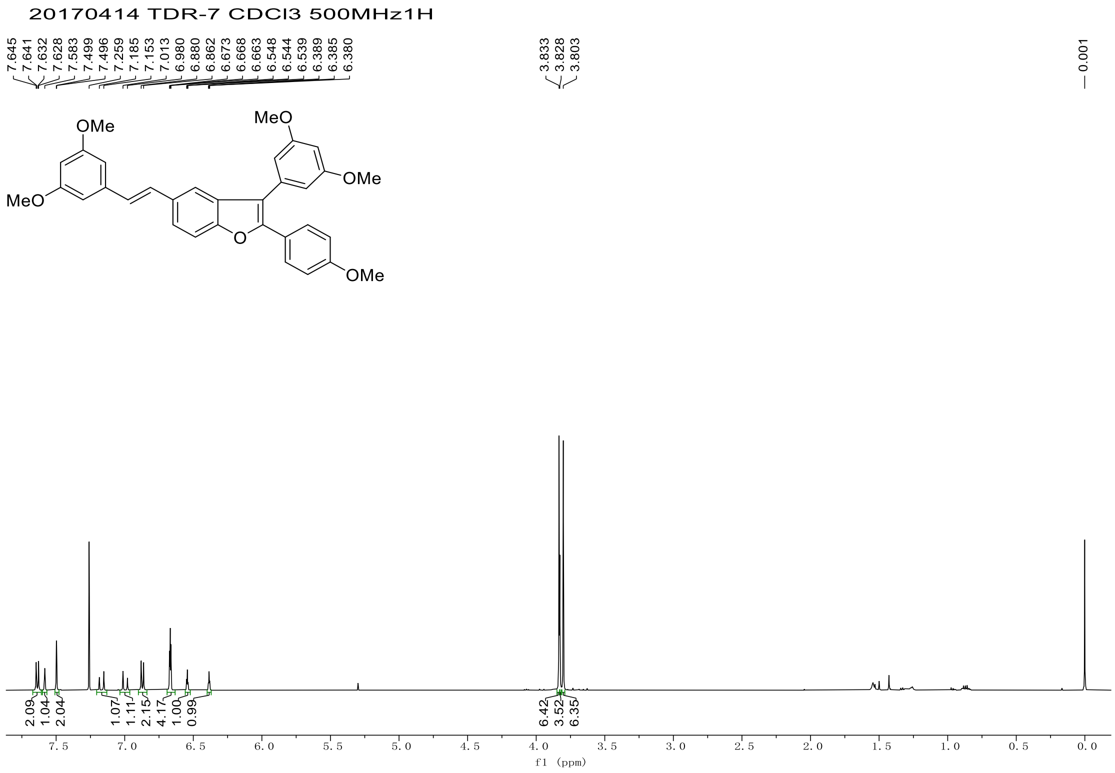

^{13}C NMR of Compound **21**

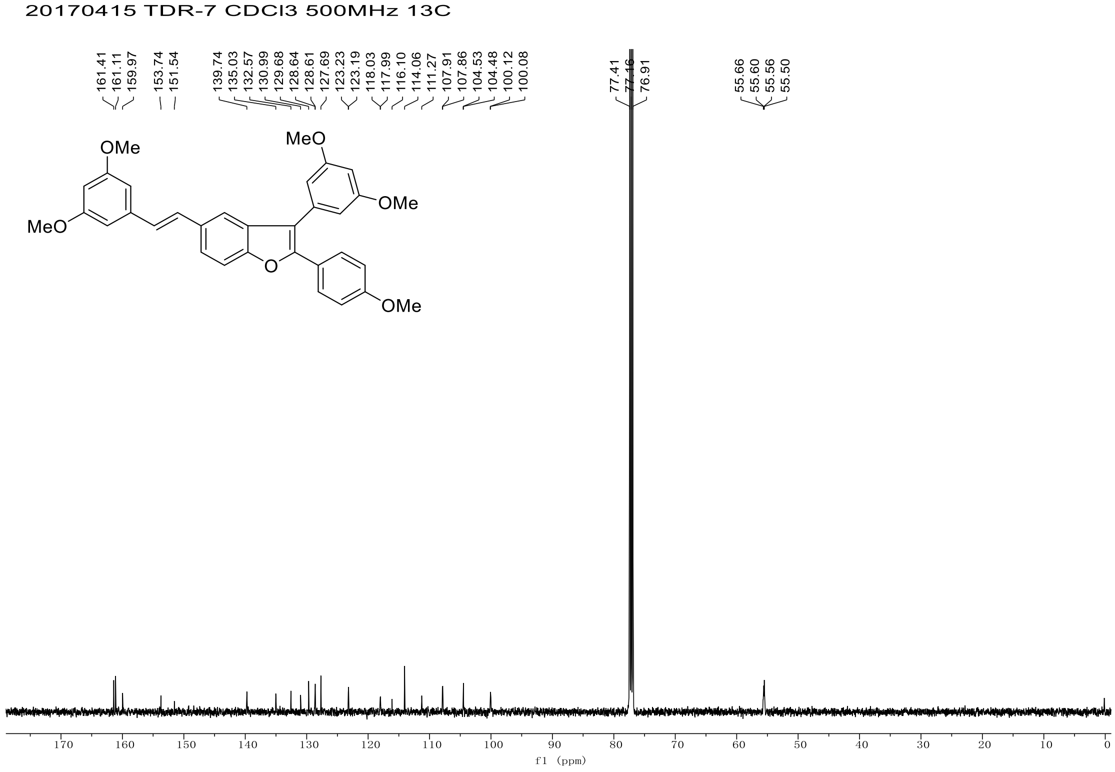

HRESI of Compound **21**

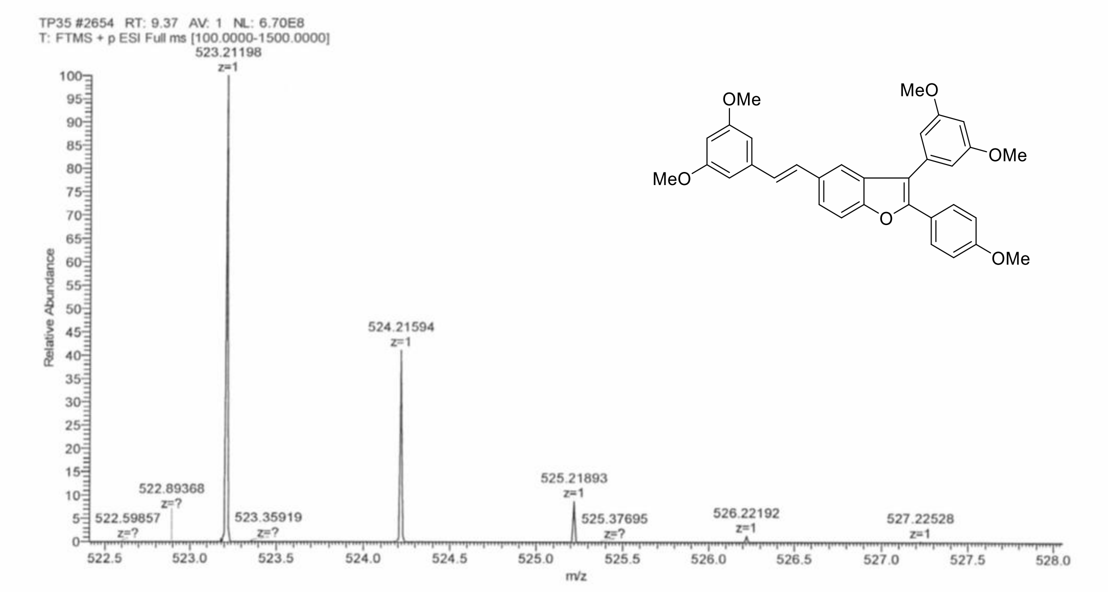

m/z	Theo. Mass	Delta (ppm)	RDB equiv.	Composition	
523.21198	523.21152	0.89	18.5	C33 H31 O6	M+H

3.13 Spectra of 1

^{1}H NMR of Compound **1**

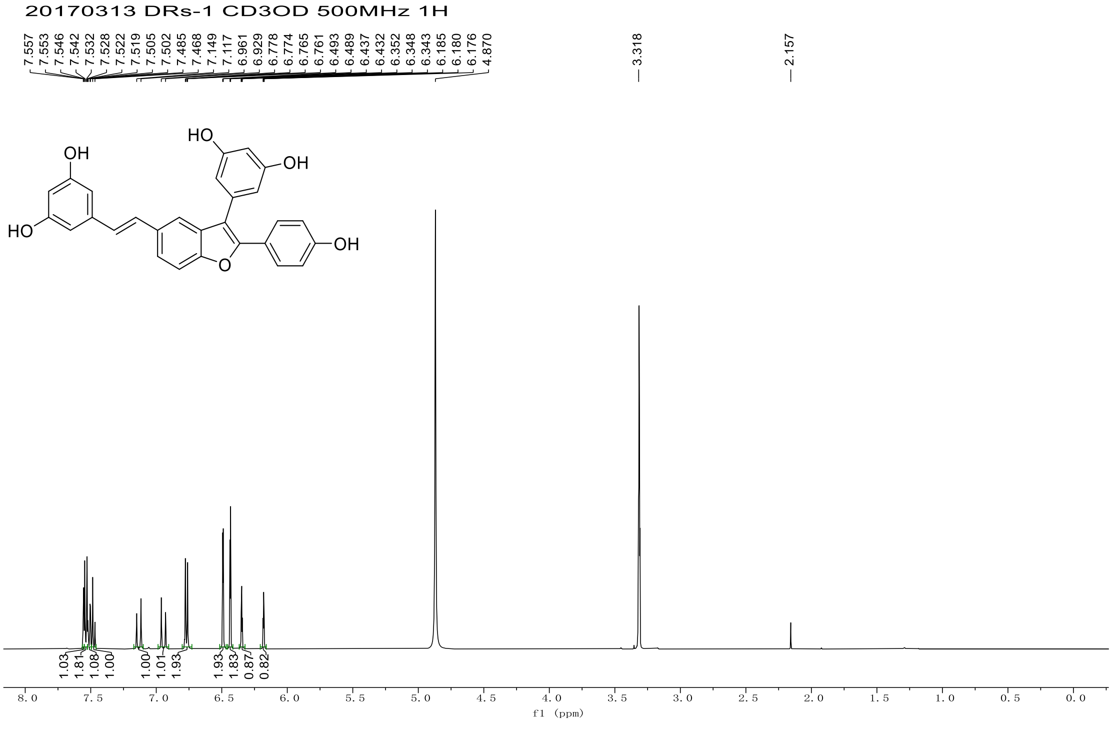

^{13}C NMR of Compound **1**

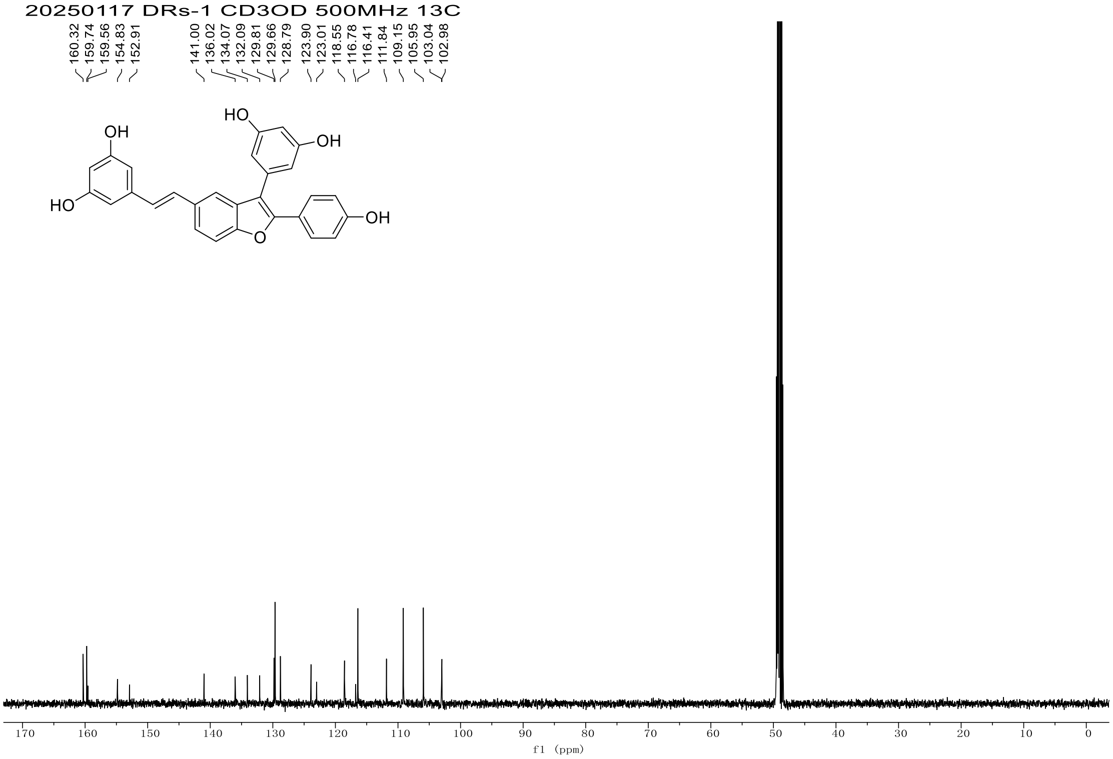

HRESI of Compound **1**

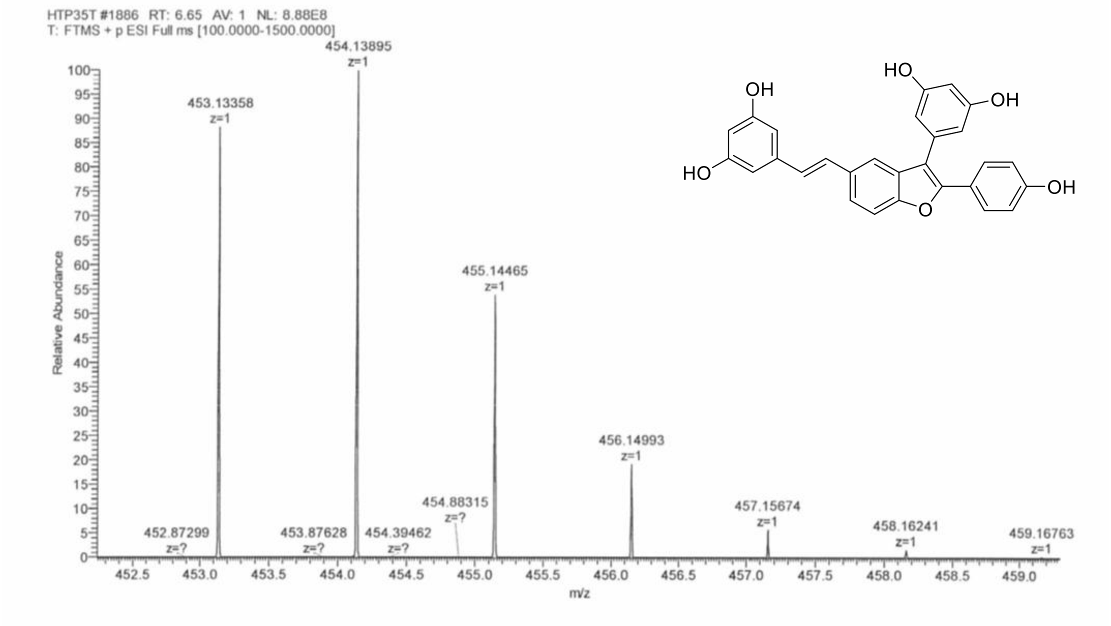

m/z	Theo. Mass	Delta (ppm)	RDB equiv.	Composition	
453.13358	453.13326	0.7	18.5	C28 H21 O6	M+H
